# ETV4 transcription factor and MMP13 metalloprotease are interplaying actors of breast tumorigenesis

**DOI:** 10.1186/s13058-018-0992-0

**Published:** 2018-07-11

**Authors:** Mandy Dumortier, Franck Ladam, Isabelle Damour, Sophie Vacher, Ivan Bièche, Nathalie Marchand, Yvan de Launoit, David Tulasne, Anne Chotteau-Lelièvre

**Affiliations:** 1 University of Lille, CNRS, Institut Pasteur de Lille, UMR 8161 - M3T – Mechanisms of Tumorigenesis and Targeted Therapies, F-59000 Lille, France; 2 Department of Biochemistry and Molecular Pharmacology, University of Massachusetts Medical School, Worcester, MA 01605-2324 USA; 3 Unit of Pharmacogenomics, Department of Genetics, Institut Curie, Paris, France; 4 CNRS UMR 8161, Institut de Biologie de Lille - Institut Pasteur de Lille, 1 Rue Pr Calmette, BP447, 59021 Lille, France

**Keywords:** ETV4, Transcription factor, MMP13, Tumorigenesis, Breast cancer

## Abstract

**Background:**

The ETS transcription factor ETV4 is involved in the main steps of organogenesis and is also a significant mediator of tumorigenesis and metastasis, such as in breast cancer. Indeed, ETV4 is overexpressed in breast tumors and is associated with distant metastasis and poor prognosis. However, the cellular and molecular events regulated by this factor are still misunderstood. In mammary epithelial cells, ETV4 controls the expression of many genes, *MMP13* among them. The aim of this study was to understand the function of MMP13 during ETV4-driven tumorigenesis.

**Methods:**

Different constructs of the *MMP13* gene promoter were used to study the direct regulation of *MMP13* by ETV4. Moreover, cell proliferation, migration, invasion, anchorage-independent growth, and in vivo tumorigenicity were assayed using models of mammary epithelial and cancer cells in which the expression of MMP13 and/or ETV4 is modulated. Importantly, the expression of *MMP13* and *ETV4* messenger RNA was characterized in 456 breast cancer samples.

**Results:**

Our results revealed that ETV4 promotes proliferation, migration, invasion, and anchorage-independent growth of the MMT mouse mammary tumorigenic cell line. By investigating molecular events downstream of ETV4, we found that MMP13, an extracellular metalloprotease, was an ETV4 target gene. By overexpressing or repressing MMP13, we showed that this metalloprotease contributes to proliferation, migration, and anchorage-independent clonogenicity. Furthermore, we demonstrated that MMP13 inhibition disturbs proliferation, migration, and invasion induced by ETV4 and participates to ETV4-induced tumor formation in immunodeficient mice. Finally, ETV4 and MMP13 co-overexpression is associated with poor prognosis in breast cancer.

**Conclusion:**

MMP13 potentiates the effects of the ETV4 oncogene during breast cancer genesis and progression.

**Electronic supplementary material:**

The online version of this article (10.1186/s13058-018-0992-0) contains supplementary material, which is available to authorized users.

## Background

ETV4, together with ETV1 and ETV5, constitutes the PEA3 group among the 12 subgroups of the ETS transcription factor family, defined by their conserved DNA binding domain (ETS binding domain) [1, 2]. They control the development of various organs and are involved in the progression of many cancers, including breast cancer [1, 3–8]. ETV4 directly influences the outcome of mammary tumorigenesis induced by the ERBB2, steroid receptor coactivator 1, and Wnt1 oncogenes [9–11]. However, the cellular and molecular mechanisms regulated by the ETV4 factor during mammary cancer progression are still poorly understood.

In most cases, carcinogenesis is associated with an overexpression of ETV4 promoting proliferation, migration, and/or invasion involved in the tumorigenic and/or metastatic process. As a consequence, deregulation of ETV4 target genes has a key role in these processes. Few ETV4 target genes involved in the regulation of these biological responses have been described so far, particularly in the mammary cells and tissues. In these latter cases, ETV4 has been shown to regulate the expression of several matrix metalloproteases (MMPs), such as MMP2 or MMP9; transcription factors involved in epithelial-to-mesenchymal transition (EMT), such as Twist1 or Snail; or other cancer-related factors, such as Bax, cyclin D3, or cyclin D2. Therefore, they play an active role during the acquisition of invasive properties by mammary cancer cells [8, 10, 12–15]. A transcriptome-wide identification of ETV4-responsive genes in mammary cells has shown that many more genes are potentially regulated by ETV4, although it is still unclear if they are direct targets and what roles they could play in the context of ETV4-driven tumorigenesis [16]. Therefore, the precise characterization of ETV4 target genes in the context of mammary tumorigenesis will allow a better understanding of the molecular mechanisms involved in this pathology.

*MMP13* is one of those genes and was identified as being downregulated following ETV4 knock-down in mammary epithelial cells [16]. MMP13 (collagenase 3) belongs to the collagenase subfamily of MMPs and degrades all fibrillary collagens, particularly the type II collagen [17]. MMP13 has a role in different kind of cancer [18] and is overexpressed in a variety of malignant tumors [19]. It was first identified from overexpressing breast carcinomas [20]. Although the role of MMP13 in mammary tumorigenesis has been reported [18, 21–27], its regulation in the oncogenic process is still misunderstood. Indeed, MMP13 is expressed in the endothelium surrounding breast tumors, suggesting a role in the modulation of extracellular matrix degradation and cell-matrix interactions involved in metastasis [20, 28]. Consistently, functional evidence demonstrates that MMP13 increases the invasive capacities of the malignant cells in breast cancer [29–31]. Yet, the precise role of the MMP13 protein and how the *MMP13* gene is transcriptionally regulated during mammary tumorigenesis remain unclear.

On the basis of the results of this study, we first report that *MMP13* is an ETV4 target gene in various mammary cellular models, and we identify an ETS binding site necessary for the direct regulation of the *MMP13* gene promoter by ETV4. Second, by establishing ETV4- and MMP13-overexpressing and MMP13-repressing MMT cells to assess modification of the phenotypic cellular properties, we show that ETV4 significantly promotes cell proliferation, migration, invasion, and anchorage-independent growth. Moreover, we provide evidence that MMP13, to a lesser extent, presents the same contribution. Next, we assess the consequences of MMP13 knock-down in ETV4-controlled events. Interestingly, MMP13 inhibition disturbs the positive effect of ETV4 on MMT proliferation, migration, and invasion, and we demonstrate that MMP13 acts as a relay of ETV4 in its functional role in the mammary epithelial tumorigenic cells in vitro as well as in tumor graft assays in vivo. Finally, we investigate the ETV4-MMP13 link in breast cancer samples and describe that the association of both ETV4 and MMP13 overexpression is associated with poor patient outcome. Ultimately, these data shed light on a new ETV4 relay, the extracellular metalloprotease MMP13, which could potentially be targeted in the context of ETV4-controlled mammary tumorigenesis.

## Methods

### Cell culture and reagents

The TAC murine mammary epithelial cell line [32, 33] was cultured on collagen-coated plates in Gibco high-glucose DMEM (Thermo Fisher Scientific, Waltham, MA, USA) supplemented with 10% FCS, penicillin (110 IU/ml), and streptomycin (110 μg/ml). A wild-type mouse mammary tumor (MMT) cell line (ATCC® CCL-51™; American Type Culture Collection, Manassas, VA, USA) was cultured in DMEM supplemented with 10% (vol/vol) FBS, gentamicin (100 IU/ml), and nonessential amino acids (Gibco; Thermo Fisher Scientific). The MCF10A cell line (ATCC® CRL-10317™; American Type Culture Collection) was propagated in DMEM/F-12 medium (Gibco; Thermo Fisher Scientific) supplemented with 5% horse serum, 20 ng/ml epidermal growth factor, 100 ng/ml cholera toxin, 500 ng/ml hydrocortisone, and 0.01 mg/ml insulin.

### Plasmids

pTracer-ETV4 and pLPCX-ETV4-V5 plasmids were described previously [8, 12, 32]. pMX-MMP13 was generated by PCR amplification of the mouse *MMP13* complementary DNA (cDNA) and cloned into the pMX-Puro retroviral vector. pRS-shMMP13 retroviral plasmid was kindly provided by S. Meierjohann [34]. pGL3 constructs containing different parts of the *MMP13* promoter were kindly provided by J. M. Davidson [35]. Mutations to proximal ETS and activator protein (AP)-1 binding site in MMP13 promoter were made using the QuikChange® II XL Site-Directed Mutagenesis Kit (Agilent Technologies, Santa Clara, CA, USA). The proximal ETS site was changed from **GG**AA to **CC**AA, and the proximal AP-1 site was changed from **TG**ACT to **GC**ACT. The sequence of all promoter constructs was verified by DNA sequencing.

### Retroviral infections and stable selection

HEK293GP packaging cells (Clontech) (3 × 10^6^) were transfected with pLPCX, pMX, or pRS retroviral constructs, and 1.2 × 10^6^ MMT cells or 1 × 10^6^ MCF-10A cells per 100-mm dish were incubated with supernatant as previously described [16]. The selection procedure was started the next day using puromycin (Life Technologies, Carlsbad, CA, USA).

### Stable cell lines

TAC cells overexpressing ETV4 after retroviral infection (ETV4) and control mock-infected TAC cells (ctrl) were previously described [8]. MMT cells infected with pLPCX retroviral vector (Ctrl) were used as a control for MMT cells overexpressing ETV4 after retroviral infection with the pLPCX-ETV4 vector (ETV4). MMT cells infected with pMX retroviral vector (Ctrl) were used as a control for MMT cells overexpressing MMP13 after retroviral infection with the pMX-MMP13 vector (MMP13).

MMT cells infected with pRS retroviral vector (shCtrl) were used as a control for MMT cells overexpressing shMMP13 after retroviral infection with the pRS-shMMP13 vector (shMMP13). MMT-ETV4 cells (overexpressing ETV4) infected with pRS retroviral vector (ETV4 + shCtrl) were used as a control for MMT-ETV4 cells overexpressing shMMP13 after retroviral infection with the pRS-shMMP13 vector (ETV4 + shMMP13).

### RNA extraction, reverse transcription, and real-time qPCR

Total RNA was extracted using the RNeasy Mini Kit according to the manufacturer’s instructions (Qiagen, Hilden, Germany). Total RNA (1 μg) was reverse-transcribed using the high-capacity cDNA reverse transcription kit (Life Technologies). Specific gene expression was determined by real-time PCR using the Fast SYBR® Green Master Mix (Life Technologies) and the Mx3005P qPCR system (Agilent Technologies). The results were analyzed with the comparative cycle threshold method normalized to cyclophilin A and compared with a comparator sample. The nucleotide sequences of the primers used were as follows: MMP13-F (5′-TCCCTGCCCCTTCCCTATGG-3′) and MMP13-R (5′-CTCGGAGCCTGTCAACTGTGG-3′) for the *MMP13* gene (PCR product of 173 bp), ETV4-F (5′-CCGCTCGCTGCGATACTATT-3′) and ETV4-R (5′-CGGTCAAACTCAGCCTTCAGA-3′) for the *ETV4* gene (PCR product of 162 bp), and PPIA-F (5′-GGGAACCGTTTGTGTTTGGT-3′) and PPIA-R (5′-TGTGCCAGGGTGGTGACTTT-3′) for *PPIA* gene.

### Luciferase reporter assays

TAC cells (3 × 10^4^) were seeded in 12-well plates and transfected with ExGen 500 (Euromedex, Strasbourg, France) and 250 ng of DNA (200 ng of expression vector, 25 ng of firefly luciferase reporter vector, 25 ng of the Renilla luciferase pRL-TK; Promega, Madison, WI, USA), according to a protocol previously described [12].

### Chromatin immunoprecipitation

TAC pLNCX, pLNCX-ETV4 and MMT pLPCX, pLPCX ETV4 were fixed, lysed, and used for chromatin immunoprecipitation (ChIP) with an anti-ETV4 immunoglobulin G (IgG) (sc-113x; Santa Cruz Biotechnology, Dallas, TX, USA) or a nonrelevant antibody normal rabbit IgG (sc-2027), as described in Additional file 1. Detection of specific DNA regions was performed by PCR using the *MMP13* gene promoter region and CCND2 gene promoter region. The nucleotide sequences of the primers used were as follows: MMP13 forward: 5′-TCCATTTCCCTCAGATTCTGCCAC-3′ and MMP13 reverse: 5′- TCTCTCCTTCCCAGGGCAAGCAT-3′ for the *MMP13* gene (PCR product of 164 bp) and CCND2 forward: 5′-GAGAGGGAGGGAAAGATTGAAAGGA-3′ and CCND2 reverse: 5′- AGGTGGGCGAGCGGAGCCTCAAG-3′ for the CCND2 gene (PCR product of 212 bp).

### RNA interference

*MMP13* and oligonucleotides used for RNA interference were purchased from Dharmacon (SMARTpool ON-TARGET*plus MMP13* J-047459, Dharmacon, Lafayette, CO, USA) and consisted of a mix of four siRNAs: J-047459-12-GGCCCAUACAGUUUGAAUA/J-04745911-AGACUAUGGACAAAGAUUA/J-047459-10-UCAAAUGGUCCCAAACGAA/J-047459-9-CUGCGACUCUUGCGGGAAU. Control oligonucleotide consisted of ON-TARGET*plus* nontargeting siRNA#1 (D-001810-01-UGGUUUACAUGUCGACUAA control siRNA). MMT cells (3.5 × 10^5^) were seeded in six-well plates for reverse transfection with 75 pmol of each siRNA and 5 μl of Lipofectamine® 2000 reagent (Thermo Fisher Scientific) as recommended by the manufacturer. Cells were incubated for another 24 hours under standard conditions before being assayed.

### Western blotting

Cells were lysed in buffer made of 150 mM NaCl, 50 mM Tris-HCl, pH 7.5, 1% Nonidet P-40 (vol/vol), 1 mM sodium orthovanadate, 1 mM phenylmethylsulfonyl fluoride, 10 g/ml leupeptin, and 10 g/ml aprotinin. After scraping, cellular debris was removed by centrifugation at 10,000 × *g* for 5 minutes. Protein concentrations were determined by using a Bradford assay. For the supernatant, a subconfluent culture was grown for 24 hours in serum-free medium, then the supernatant was centrifuged at 3000 × *g* for 3 minutes. Whole-cell extracts (50 μg) or 1:20 of supernatants were separated in precast gels (Mini-PROTEAN® TGX Stain-Free™; Bio-Rad Laboratories, Hercules, CA, USA) gel and transferred onto nitrocellulose membranes (Trans-Blot Turbo Transfer System; Bio-Rad Laboratories). After blocking with Tris-buffered saline, 0.1% Tween, and 3% bovine serum albumin (BSA), the membrane was probed with the primary and secondary antibodies. The enzymatic activity was detected using an Amersham enhanced chemiluminescence kit (GE Healthcare Life Sciences, Marblehead, MA, USA). Equal transfer of proteins from the gel was controlled by using the stain-free system of the gel and the membrane as well as by using an anti-GAPDH antibody. We used anti-ETV4 1:500 (GTX114393; GeneTex, Irvine, CA, USA), anti-MMP13 1:500 (18165-1-AP; Proteintech, Rosemont, IL, USA), anti-GAPDH 1:1000 (6C5-sc-32233; Santa Cruz Biotechnology), and secondary antimouse or antirabbit antibodies coupled to horseradish peroxidase (HRP) (GE Healthcare Life Sciences).

### Zymography

Gelatin zymography was used to determine the activity of MMP13. The supernatant from subconfluent serum-free culture medium was collected, and the cells were removed by centrifugation. Then, 40 μl of sample were loaded onto a 10% precast polyacrylamide gel with 0.1% of gelatin (Bio-Rad Laboratories). After electrophoresis, the gels were renatured by soaking for 30 minutes at room temperature in 2.5% Triton X-100. The gels were then incubated in a developing buffer (50 mM Tris, 200 mM NaCl, 5 mM CaCl_2_, 0.02% Brij-35 [MilliporeSigma], pH 7.5) overnight at 37 °C. The gels were stained with Coomassie Brilliant Blue R-250 and destained in demineralized water. The transparent bands of gelatinolytic activity were visualized as clear bands against the blue-stained gelatin background.

### Cell proliferation assays

Stable MMT cells (1.5 × 10^4^; pLPCX/pLPCX-ETV4-pMX/pMX-MMP13-pRS/pRS-shMMP13 and MMT-ETV4-pRS/MMT-ETV4-pRS-shMMP13) were seeded in six-well plates. The cells were trypsinized and counted after 10, 35, 55, 80, and 100 hours using an Invitrogen Tali™ Image-based Cytometer (Thermo Fisher Scientific). Each time point was counted three times.

### Anchorage-independent growth

Stable MMT cells (3 × 10^5^; pLPCX/pLPCX-ETV4-pMX/pMX-MMP13-pRS/pRS-shMMP13 and MMT-ETV4-pRS/MMT-ETV4-pRS-shMMP13) were seeded in 500 μl of medium mixed with 1 ml of 0.35% agar in growth medium (DMEM [Thermo Fisher Scientific] with 10% FBS). The cell suspension was cast onto 12-well plates with 500 μl of 0.65% agar in growth medium, which was used as an underlay. Growth medium was added onto the agar layer and changed weekly. Colonies were photographed after 10 days using a light microscope (Axio Vert A.1; Carl Zeiss Microscopy, Jena, Germany).

### Cell migration assays

Boyden chamber cell migration was assayed using a cell culture chamber insert system (BD Biosciences, San Jose, CA, USA) with an 8-μm polyethylene terephthalate (PET) membrane. Stable MMT cells (4 × 10^4^; pLPCX/pLPCX-ETV4-pMX/pMX-MMP13-pRS/pRS-shMMP13 and MMT-ETV4-pRS/MMT-ETV4-pRS-shMMP13) were seeded in the upper chamber in DMEM with 10% FBS. The same medium was added in the lower chamber. After 18 hours, cells that did not cross the membrane were scraped off the upper side of the membrane with a cotton swab. Cells that had migrated to the lower side were fixed with methanol at − 20 °C and stained with Hoechst 33258 (MilliporeSigma). The membrane was excised from its support and mounted on a glass side with Dako Glycergel mounting medium (Agilent Technologies). Cells were photographed using a light microscope (Axio Vert A.1) and counted using ImageJ software (National Institutes of Health, Bethesda, MD, USA).

### Cell invasion assays

Boyden chamber cell invasion was assayed using a cell culture chamber insert system (Corning® BioCoat™ Growth Factor Reduced Matrigel® Invasion Chamber; Corning Life Sciences, Corning, NY, USA) with an 8-μm PET membrane coated with Matrigel®. Stable MMT cells (8 × 10^4^; pLPCX/pLPCX-ETV4-pMX/pMX-MMP13-pRS/pRS-shMMP13 and MMT-ETV4-pRS/MMT-ETV4-pRS-shMMP13) were seeded in the upper chamber in DMEM with 0% FBS and 0.1% BSA. DMEM with 5% FBS was added in the lower chamber. After 36 hours, cells that did not cross the membrane were scraped off the upper side of the membrane with a cotton swab. Cells that had migrated to the lower side were fixed with methanol at − 20 °C and stained with Hoechst 33258. The membrane was excised from its support and mounted on a glass side with Dako Glycergel mounting medium. Cells were photographed using a light microscope (Axio Vert A.1) and counted using ImageJ software.

### Tumor grafts

MMT pLPCX-ETV4-pRS and MMT pLPCX-ETV4-pRS-shMMP13 cells were trypsinized, then suspended in PBS (5 × 10^6^ cells/ml). Cells (5 × 10^5^) were injected subcutaneously into the inguinal flank of 6–7-week-old female severe combined immunodeficiency (SCID)-deficient mice. A total of six mice per condition were used in three independent experiments. Tumor size was assessed by measuring the length and width of tumors every 3–4 days. Tumor volume was estimated using the formula: (length × width^2^)/2. The experiments were stopped when the largest tumors reached the critical size of about 10% of the mouse’s weight in accordance with the ethical approval form, thus meaning between 2 and 3 weeks postinjection. At the time the mice were killed, the tumors were removed, fixed in 4% paraformaldehyde, and embedded in paraffin. Results are expressed as the mean tumor volume for each experimental group. All animal procedures were conducted with the approval of and in compliance with the guidelines of the Nord Pas de Calais Regional for Ethical Animal Care and Use Committee (CEEA-003243.01).

### IHC

IHC of paraffin-embedded mouse tumor tissue sections was performed using anti-ETV4 (1:100, GTX100812; GeneTex), anti-MMP13 (1:100, 18165-1-AP; Proteintech), anti-Ki67 (1:200, ab15580; Abcam, Cambridge, UK), and anti-cleaved caspase 3 (1:100, Asp175, catalogue no. 9661; Cell Signaling Technology, Danvers, MA, USA). Sections were incubated with secondary HRP-conjugated antibody. Counterstaining was performed using Mayer’s hematoxylin (Merck, Darmstadt, Germany). Imaging was carried out using ZEN Blue imaging software (Carl Zeiss Microscopy).

### Patients and samples for MMP13 and ETV4 expression

Samples of 456 primary unilateral invasive breast tumors excised from women managed at Curie Institute-René Huguenin Hospital (St. Cloud, France) from 1978 to 2008 were analyzed. Immediately after biopsy or surgery, the tumor samples were stored in liquid nitrogen until messenger RNA (mRNA) extraction. Tumor samples were considered suitable for our study if the proportion of tumor cells exceeded 70%.

All patients (mean age 61.7 years, range 31–91 years) met the following criteria: primary unilateral nonmetastatic breast carcinoma for which complete clinical, histological, and biological data were available; no radiotherapy or chemotherapy before surgery; and full follow-up at Curie Institute-René Huguenin Hospital. Treatment consisted of modified radical mastectomy in 278 cases (63.6%) and breast-conserving surgery plus locoregional radiotherapy in 159 cases (36.4%) (information available for only 437 cases). The patients underwent a physical examination and routine chest radiography every 3 months for 2 years, then annually. Mammograms were done annually. Adjuvant therapy was administered to 369 patients, consisting of chemotherapy alone in 91 cases, hormone therapy alone in 176 cases, and both treatments in 102 cases. The histological type and the number of positive axillary nodes were established at the time of surgery. The malignancy of infiltrating carcinomas was scored according to the Scarff-Bloom-Richardson (SBR) histoprognostic grading system. Hormone receptor (HR) [estrogen receptor (ERα), progesterone receptor (PR)] and human epidermal growth factor receptor 2 (ERBB2) status were determined at the protein level by using biochemical methods (dextran-coated charcoal method, enzyme immunoassay, or IHC) and confirmed by qPCR assays as described in Additional file 1.

The population was divided into four groups according to HR (ERα and PR) and ERBB2 status, as follows: two luminal subtypes [HR+ (ERα + or PR+)/ERBB2+ (*n* = 54)] and [HR+ (ERα + or PR+)/ERBB2− (*n* = 289)]; an ERBB2+ subtype [HR− (ERα− and PR−)/ERBB2+ (*n* = 45)] and a triple-negative subtype [HR− (ERα− and PR−)/ERBB2− (*n* = 68)]. The median follow-up was 8.9 years (range 130 days to 33.2 years). One hundred eighty-one patients had a metastasis. Clinicopathological characteristics of patients in relation to metastasis-free survival (MFS) are provided in Additional file 2: Table S1. Ten specimens of adjacent normal breast tissue from patients with breast cancer or normal breast tissue from women undergoing cosmetic breast surgery were used as sources of normal mRNA.

### Statistical analysis

For in vitro and in vivo analyses, all values are expressed the means of triplicate samples ± SE. Data were analyzed using unpaired *t* tests.

For human statistical analysis, relationships between mRNA expression of genes and clinical parameters were identified using nonparametric tests, the χ^2^ nonparametric test (relationship between two qualitative parameters), and the Kruskal-Wallis H test (relationship between one quantitative parameter and two or more qualitative parameters). Differences were considered significant at confidence levels greater than 95% (*P* < 0.05). MFS was determined as the interval between initial diagnosis and detection of the first metastasis.

To visualize the efficacy of *MMP13* and *ETV4* mRNA levels for discriminating between two populations (patients who developed/did not develop metastases) in the absence of an arbitrary cutoff value, data were summarized in an ROC curve. The AUC was calculated as a single measure to discriminate efficacy. The population was divided into four patient subgroups according to *MMP13* ROC curve value in the series of 456 breast cancer samples and then according to *ETV4* ROC curve value in the high and low *MMP13* mRNA expression level subpopulations. Finally, the very small subgroup (*n* = 16) of high *MMP13*/low *ETV4* mRNA expression level was merged with the subgroup (*n* = 66) of low *MMP13*/low *ETV4* mRNA expression level to obtain a unique group of 82 patients with low *ETV4* mRNA expression level. Survival distributions were estimated by the Kaplan-Meier method, and the significance of differences between survival rates was ascertained with the log-rank test. The Cox proportional hazards regression model was used to assess prognostic significance in multivariate analysis [36]. We also analyzed an independent dataset of breast tumors for which microarray data were publicly available (Netherlands Cancer Institute [NKI], *n* = 295; http://ccb.nki.nl/data/).

## Results

### *MMP13* is an ETV4 target gene in mammary epithelial cells

**Fig. 1:**
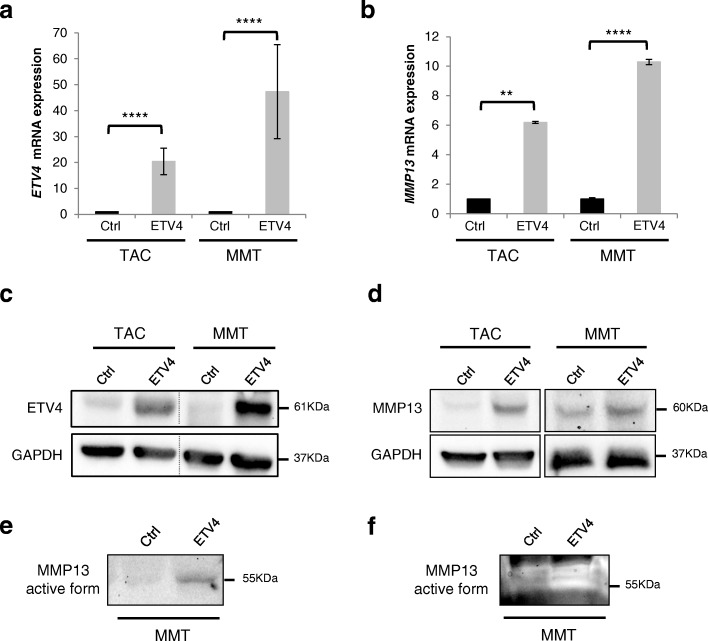
Expression of ETV4 and MMP13 in TAC-Ctrl/ETV4 and MMT-Ctrl/ETV4 cells. **a** and **b** Relative *ETV4* (**a**) or *MMP13* (**b**) mRNA expression in TAC/MMT-Ctrl and TAC/MMT-ETV4-overexpressing cells determined by real-time PCR and normalized to cyclophilin A levels. mRNA expression in TAC/MMT-Ctrl cells was arbitrarily = 1. Error bars indicate SD. ****P ≤ 0.0001; **P ≤ 0.01. **c** and **d** Western blot analysis of ETV4 protein expression (61 kDa) (**c**) or MMP13 protein expression (60 kDa) (**d**) in TAC/MMT-Ctrl and TAC/MMT-ETV4 cells. GAPDH expression served as the loading control. **e** Western blot analysis of the secreted MMP13 protein expression (55 kDa) from the supernatant of MMT-Ctrl and MMT-ETV4-overexpressing cells. **f** Zymographic analysis of MMP13 protein activity (55 kDa) in MMT-Ctrl and MMT-ETV4 cells

We previously described ETV4-regulated genes in mammary tumorigenic MMT cells following ETV4 inhibition [16]. Among them, MMPs such as MMPs 1, 2, 3, 9, and 14 were shown to be slightly regulated [16]. However, our attention was focused on MMP13, which was identified as a potentially interesting ETV4 target gene through large-scale transcriptomic analysis that we performed on these MMT cells [16], thereafter completed with transcriptomic analysis performed with ETV4-overexpressing and ETV4-repressing TAC cells (unpublished data). In these latter analyses, we found that ETV4 positively modulates *MMP13* gene expression (18.12-fold, *P* = 0.0014 following ETV4 overexpression; 0.16-fold, *P* = 0.02 following ETV4 inhibition). In order to characterize the regulation of *MMP13* expression by ETV4, we used the mammary epithelial TAC cell line overexpressing ETV4 [8] as well as the mammary cancerous MMT cell line and the breast epithelial MCF10A cell line, engineered to overexpress a V5-tagged ETV4 protein after a retroviral infection (MMT-ETV4 and MCF10A-ETV4) (Fig. 1a and c and Additional file 3: Figure S1a and c). Overexpression of ETV4 upregulates *MMP13* mRNA and protein expression in the mouse TAC, MMT (Fig. 1b and d) and human MCF10A cells (Additional file 3: Figure S1b and d). Moreover, the secretion of the active form of MMP13 is increased in the supernatant of ETV4-overexpressing cells, as shown by Western blotting (Fig. 1e) and zymography (Fig. 1f).

**Fig. 2:**
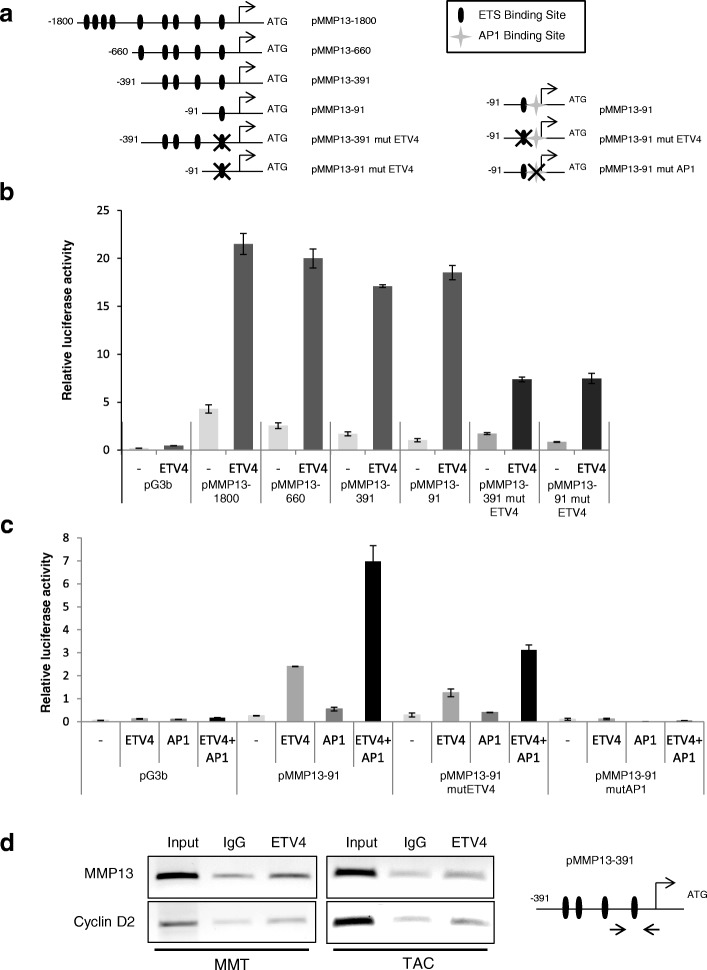
*MMP13* gene is an ETV4 target gene in mammary epithelial TAC and MMT cells. Effect of ETS/AP1 binding site mutations in the *MMP13* promoter regulation. **a** Schematic representation of the mouse *MMP13* promoter fragments (pMMP13–1800 to pMMP13–91) and AP-1 and/or ETV4 mutant versions cloned into a pG3bLuc reporter vector (pG3b). Position of the conserved ETS binding sites (EBS) is represented by . AP-1 binding site is represented by . : Transcription start site. : Mutation of the ETS site. **b** and **c** Histograms representing the relative luciferase activity measured for each promoter construct cotransfected into the TAC cell line with pTracer vector (−) or pTracer-ETV4 expression vector (ETV4) and/or AP-1 expression vector (AP1). Experiments were conducted three times in triplicate. Error bars indicate SD. **d** ChIP experiment. PCR detection of the *MMP13* promoter region after ETV4 immunoprecipitation in MMT (left panel) and TAC (right panel). Primers allowing the amplification of the proximal *MMP13* promoter region containing EBS are schematized in the lower panel. Cyclin D2 was used as a positive control [8]. Immunoprecipitation with a nonrelevant antibody (IgG) was used as a negative control.

We next completed these data by analyzing the ETV4-regulated *MMP13* promoter. TAC cells were transfected with various *MMP13* gene promoter fragments cloned into a luciferase reporter vector and ETV4 expression vector or control vector. Our results indicate that the *MMP13* promoter (− 1800) is active in TAC cells. Moreover, ETV4 transactivates the *MMP13* gene promoter region spanning 1800 bp downstream from the translation start codon (MMP13; − 1800) (Fig. 2a and b). To delineate the responsive elements driving the promoter activity, we tested various deletion constructs (Fig. 2a) and identified a region of 91 bp (MMP13–91), which displays an optimal promoter activity as well as induction by ETV4 (Fig. 2b). This region contains a putative ETS binding site (EBS) and a putative AP-1 binding site. These two sites are very highly conserved among mouse, human, and rabbit [37, 38] (Additional file 4: Figure S2), and the EBS was previously described to be important in *MMP13* gene promoter activity [38]. In fact, the mutation of the EBS site in the MMP13 − 91 and MMP13 − 391 fragments reduced by half the transactivation by ETV4 (Fig. 2b). Moreover, AP-1 synergized the ETV4-induced transactivation effect, and the AP-1 site is required for this activity (Fig. 2c). We thereafter evidenced ETV4 recruitment to this chromatin region in TAC and MMT cells by ChIP using an antibody directed against ETV4, and, as a positive control, we analyzed the binding of ETV4 at the cyclin D2 promoter, as previously described [8] (Fig. 2d). It is noteworthy that the same results were obtained in TAC and MMT cells that overexpress ETV4 (Additional file 5: Figure S3). Therefore, *MMP13* is an ETV4 target gene in TAC and MMT mammary epithelial cells with AP-1 as a likely coactivator.

### ETV4 enhances cell proliferation, migration, invasion, and anchorage-independent growth

**Fig. 3:**
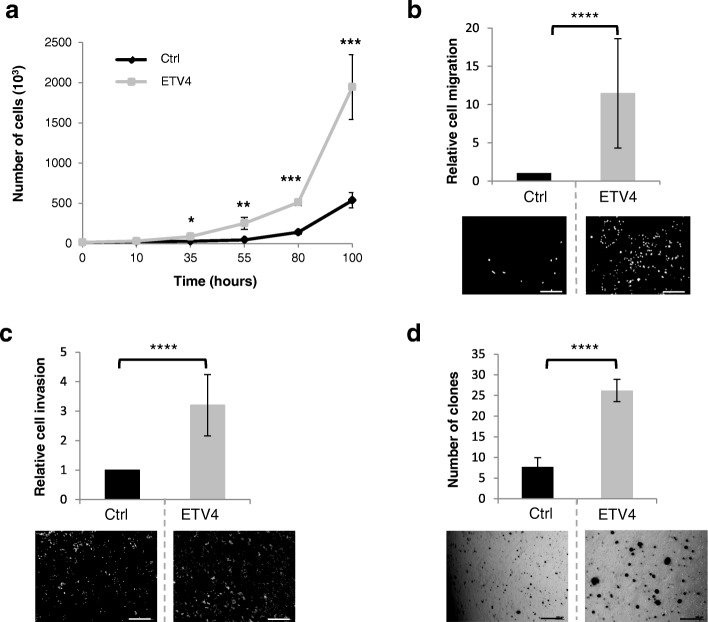
ETV4 enhances proliferation, migration, invasion, and anchorage-independent growth capacity of MMT mammary cancer cells. **a** MMT-Ctrl and MMT-ETV4 cell proliferation analysis by cell counting. The two charts represent the number of counted cells at 10, 35, 55, 80, and 100 hours. Experiments were conducted three times in triplicate. Error bars indicate SD. ***P ≤ 0.001; **P ≤ 0.01; *P ≤ 0.1. **b** MMT-Ctrl and MMT-ETV4 cell migration analysis using a Boyden chamber culture system. Histograms represent the relative number of counted cells that migrated to the lower side. The number of MMT-Ctrl cells was arbitrarily = 1. Experiments were conducted three times in triplicate. Error bars indicate SD. ****P ≤ 0.0001. The lower panel depicts a representative picture of each experiment. Scale bar = 100 μm. **c** MMT-Ctrl and MMT-ETV4 cell invasion analysis using a Boyden chamber culture system coated with Matrigel®. Histogram represents the relative number of cells that invaded to the lower side. The number of MMT-Ctrl cells was arbitrarily = 1. Experiments were conducted three times in triplicate. Error bars indicate SD. ****P ≤ 0.0001. The lower panel depicts a representative picture of each experiment. Scale bar = 100 μm. **d** Anchorage-independent growth. MMT-Ctrl and MMT-ETV4 cells were cultured for 10 days in soft agar. This histogram represents the number of clones counted for experimental time point. Soft agar assays were conducted three times in triplicate. Magnification × 5. Error bars indicate SD. ****P ≤ 0.0001. The lower panel depicts a representative picture of each experiment. Scale bar = 100 μm

Proliferation and migration assays showed that MMT cells overexpressing ETV4 display enhanced proliferation and migration abilities as determined using a Boyden chamber (Fig. 3a and b). Similar results were obtained with or without treatment with Mitomycin C, an inhibitor of proliferation, indicating that the effect on cell migration was not a consequence of an increase in cell number (data not shown). Moreover, invasion assay in a Matrigel®-overlaid Boyden chamber and in a clonogenic assay revealed that ETV4 significantly increases invasion (Fig. 3c) and anchorage-independent growth (Fig. 3d) in vitro*.* These data confirm that ETV4 is an important actor of the cellular abilities (proliferation, migration, invasion, anchorage-independent growth) involved in the tumorigenic properties of MMT cells.

### MMP13 is a regulator of ETV4-dependent tumorigenic properties in mammary cancer cells

**Fig. 4:**
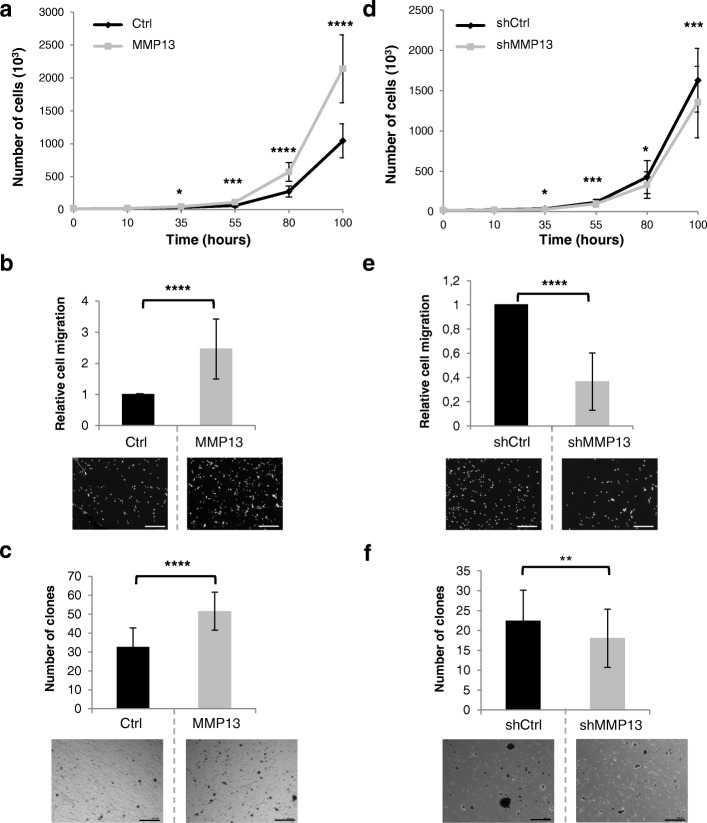
MMP13 acts as ETV4 in the modification of tumorigenic properties of MMT cells. **a** and **d** MMT-Ctrl and MMT-MMP13 (**a**) or MMT-shCtrl and MMT-shMMP13 (**d**) cell proliferation analysis by cell counting. The two charts represent the number of counted cells at 10, 35, 55, 80, and 100 hours. Experiments were conducted three times in triplicate. Error bars indicate SD. ****P ≤ 0.0001; ***P ≤ 0.001; *P ≤ 0.1. **b** and **e** MMT-Ctrl and MMT-MMP13 (**b**) or MMT-shCtrl and MMT-shMMP13 (**e**) cell migration analysis using a Boyden chamber culture system. Histogram represents the relative number of counted cells that migrated to the lower side. The number of MMT-Ctrl (**b**) and MMT-shCtrl (**e**) cells was arbitrarily = 1. Experiments were conducted three times in triplicate. Error bars indicate SD. ****P ≤ 0.0001. The lower panel depicts a representative picture of each experiment. Scale bar = 100 μm. **c** and **f** Anchorage-independent growth. MMT-Ctrl and MMT-MMP13 (**c**) or MMT-shCtrl and MMT-shMMP13 (**f**) cells were cultured for 10 days in soft agar. This histogram represents the number of clones counted for experimental time points. Soft agar assays were conducted three times in triplicate. Magnification × 5. Error bars indicate SD. ****P ≤ 0.0001; **P ≤ 0.01. The lower panel depicts a representative picture of each experiment. Scale bar = 100 μm

Next, we evaluated the role of MMP13 during MMT cell migration, invasion, or clonogenicity by using MMT cells in which MMP13 is overexpressed (MMT-MMP13) or knocked down by shRNA (MMT-shMMP13). MMP13 overexpression or repression was confirmed by qPCR (Additional file 6: Figure S4a and b), Western blotting (Additional file 6: Figure S4c) and/or zymography (Additional file 6: Figure S4d). As shown in Fig. 4, MMP13 overexpression increases cell proliferation (Fig. 4a), migration (Fig. 4b), and anchorage-independent growth (Fig. 4c). As expected, MMP13 repression leads to a reduction in cell proliferation (Fig. 4d), cell migration (Fig. 4e), and anchorage-independent cell growth (Fig. 4f). Thus, similarly to ETV4, but with a weaker effect, MMP13 is an inducer of cancer cell proliferation, migration, and invasion.

**Fig. 5:**
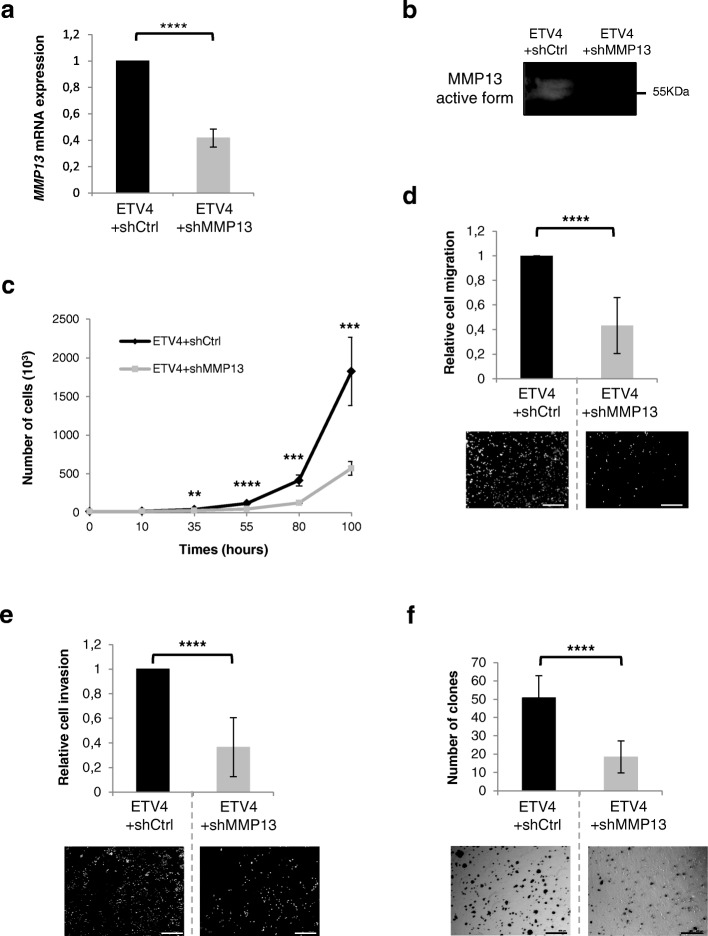
MMP13 acts as a regulator of ETV4 tumorigenic-induced response in mammary epithelial MMT cells. **a** Relative *MMP13* mRNA expression in the MMT-ETV4 + shCtrl and MMT-ETV4 + shMMP13 cells determined by real-time PCR and normalized to cyclophilin A levels. mRNA expression in MMT-ETV4 + shCtrl cells was arbitrarily = 1. Error bars indicate SD. ****P ≤ 0.0001. **b** Zymographic analysis of MMP13 protein activity (55 kDa) from the supernatant of MMT-ETV4 + shCtrl and MMT-ETV4 + shMMP13 cells. **c** MMT-ETV4 + shCtrl and MMT-ETV4 + shMMP13 cell proliferation analysis by cell counting. The two charts represent the number of counted cells at 10, 35, 55, 80, and 100 hours. Experiments were conducted three times in triplicate. Error bars indicate SD. ****P ≤ 0.0001; ***P ≤ 0.001; **P ≤ 0.01. **d** MMT-ETV4 + shCtrl and MMT-ETV4 + shMMP13 cell migration analysis using a Boyden chamber culture system. Histogram represents the relative number of counted cells that migrated to the lower side. The number of MMT-ETV4 + shCtrl cells was arbitrarily = 1. Experiments were conducted three times in triplicate. Error bars indicate SD. ****P ≤ 0.0001. The lower panel depicts a representative picture of each experiment. Scale bar = 100 μm. **e** MMT-ETV4 + shCtrl and MMT-ETV4 + shMMP13 cell invasion analysis using a Boyden chamber culture system coated with Matrigel®. Histograms represent the relative number of counted cells that invaded to the lower side. The number of MMT-ETV4 + shCtrl cells was arbitrarily = 1. Experiments were conducted three times in triplicate. Error bars indicate SD. ****P ≤ 0.0001. The lower panel depicts a representative picture of each experiment. Scale bar = 100 μm. **f** Anchorage-independent growth. MMT-ETV4 + shCtrl and MMT-ETV4 + shMMP13 cells were cultured for 10 days in soft agar. This histogram represents the number of clones counted for experimental time points. Soft agar assays were conducted three times in triplicate. Magnification × 5. Error bars indicate SD. ****P ≤ 0.0001. The lower panel depicts a representative picture of each experiment. Scale bar = 100 μm

In order to determine if MMP13 participates in ETV4-regulated cancer cell properties, we repressed MMP13 in the ETV4-overexpressing MMT cell line. To that end, we established the MMT-ETV4 + shMMP13 cell line, which expresses an MMP13-shRNA construct allowing for a significant reduction in *MMP13* mRNA expression and subsequently a reduction in MMP13 metalloprotease activity (Fig. 5a and b). Importantly, as determined by qPCR and Western blotting, ETV4 mRNA and protein expression remains unchanged in these cells (Additional file 7: Figure S5a and b). The repression of MMP13 in the ETV4-overexpressing MMT cells drastically decreases their proliferation (by 60% at 100 hours after the beginning of the experiment) (Fig. 5c) and significantly reduces cell migration (twofold decrease compared with ETV4 + shCtrl) (Fig. 5d), cell invasion (twofold decrease compared with ETV4 + shCtrl) (Fig. 5e), and anchorage-independent growth (2.5-fold increase compared with ETV4 + shCtrl) (Fig. 5f). This was confirmed by transient transfection of a siRNA directed against MMP13 in the MMT-ETV4-overexpressing cells, which led to a 60% decrease in MMP13 expression (Additional file 8: Figure S6a) and a significant reduction in anchorage-independent cell growth (Additional file 8: Figure S6b). Altogether, these results show that MMP13 acts as a relay of ETV4 to control mammary cancer cells’ tumorigenic abilities.

### MMP13 silencing inhibits the tumorigenic activity of ETV4 in vivo

**Fig. 6:**
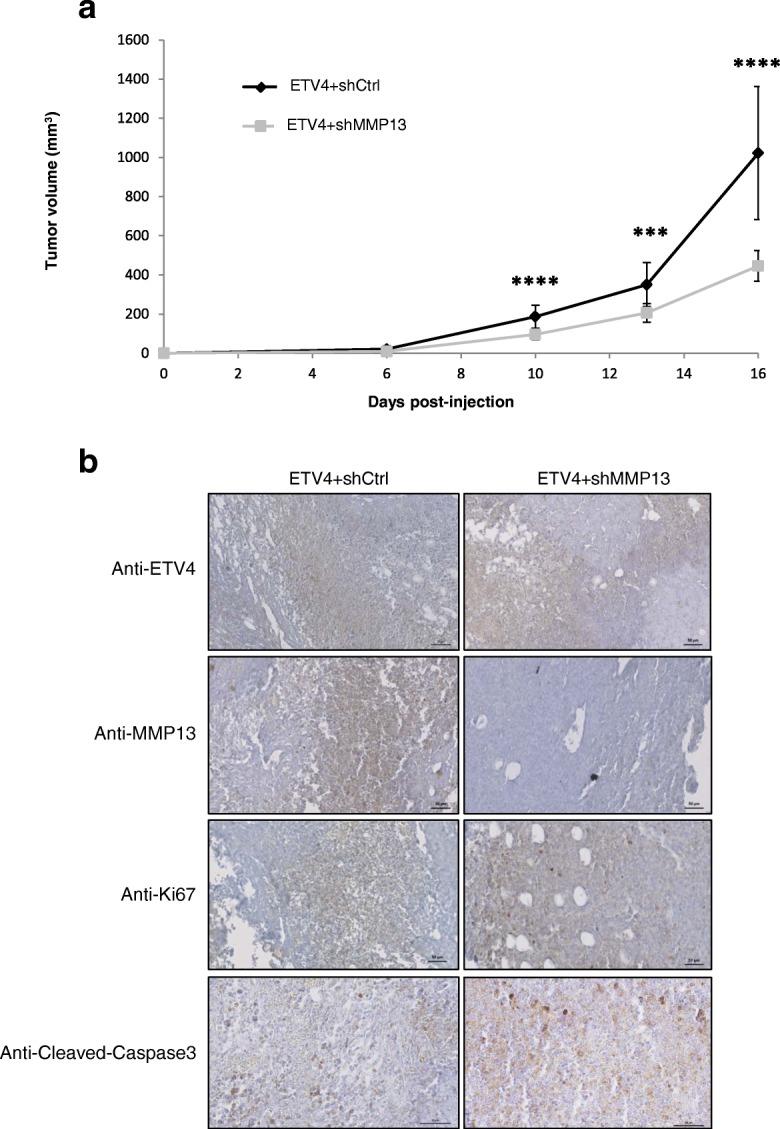
MMP13 reinforces the tumorigenic activity of ETV4 in vivo. **a** In vivo tumor growth assay. Tumor presence was determined by palpation of the mammary gland every 3–4 days. The graph represents the volume of tumor (mm^3^) versus time in weeks after graft of MMT-ETV4 + shCtrl (*n* = 14) and MMT-ETV4 + shMMP13 (*n* = 15) cells into the fat pad of the mammary gland of SCID-deficient mice. Three independent experiments were conducted. ***P ≤ 0.001 and ****P ≤ 0.0001. **b** Histologic analysis of ETV4 + shCtrl MMT cell-derived tumors (left panel) and ETV4 + shMMP13 MMT cell-derived tumors (right panel) with anti-ETV4 antibody, anti-MMP13 antibody, anti-Ki67 antibody, and anti-cleaved caspase 3 antibody. Representative staining is shown for each experiment. Scale bar = 50 μm

To investigate whether MMP13 expression is necessary for the induction of tumors by ETV4 in vivo, MMT-ETV4 + shCtrl and MMT-ETV4 + shMMP13 cells were injected into the inguinal flanks of immunocompromised mice, and tumor growth was evaluated every 3–4 days (Fig. 6). Three days postinjection, all of the mice that received an injection of MMT-ETV4 + shCtrl cells showed a palpable tumor, whereas none could be detected at this stage in the group that received an injection of MMT-ETV4 + shMMP13 cells. By day 6 postinjection, all MMT-injected mice developed palpable tumors. However, a 3–4-day measurement of tumor size over the course of 10 more days (until animals were killed) indicated that MMP13 expression was required for optimal tumor growth because MMP13-shRNA-expressing ETV4 cells are, on average, twofold smaller than controls. Immunocytochemistry performed on paraffin-embedded mouse tumor tissue sections showed an equivalent Ki-67 expression in tumors from ETV4 + shCtrl and ETV4 + shMMP13 cells, which all show proliferative activity. Cleaved caspase 3 expression showed that apoptosis events are present in both conditions, to a slightly greater extent in the ETV4 + shMMP13 tumors, according to their slow growth. ETV4 expression is, as expected, equivalent in both ETV4-expressing cell-derived tumors. In contrast, MMP13 expression decreased in ETV4 + shMMP13-derived tumors, thus confirming the suitable MMP13 regulation (here a repression) in these in vivo assays (Fig. 6b). Therefore, these data bring out that MMP13 is a mediator of ETV4 tumorigenic activity in MMT cancer cells.

### MMP13 and ETV4 expression in breast tumors is associated with a poor prognosis

**Fig. 7:**
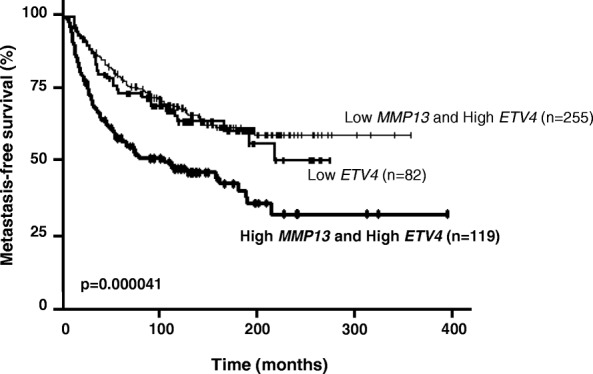
MMP13 and ETV4 are associated with poor prognosis in breast cancer. Metastasis-free survival (MFS) curves for patients with breast tumors according to Low-*ETV4* (*n* = 82), High-*ETV4* and Low-*MMP13* (*n* = 255), or High-*ETV4* and High-*MMP13* (*n* = 119) mRNA levels. ****P ≤ 0.0001

In order to corroborate the relevance of the phenotypic and mouse in vivo data and to explore the link between ETV4 and MMP13 in human breast cancer, we assessed *MMP13* and *ETV4* mRNA expression levels in a series of 456 primary unilateral invasive primary breast tumors from patients with known clinical and pathological status and long-term outcome. We used a log-rank test to identify relationships between MFS and *MMP13* and/or *ETV4* expression. Tumors with the highest levels of *MMP13* mRNA (*n* = 135 [29.6%]) were significantly associated with poor MFS (*P* = 0.00016), which was not the case for ETV4-expressing tumors (Additional file 9: Figure S7a and b). This result was confirmed in the NKI breast cancer cohort (Additional file 10: Figure S8b and c). Combined analysis (as described in the “Patients and samples for MMP13 and ETV4 expression” subsection of the Methods section above) of *MMP13* and *ETV4* mRNA expression levels defined three separate prognostic groups of 82 (Low-ETV4), 255 (High-ETV4/Low-MMP13), and 119 (High-ETV4/High-MMP13) patients with significantly different survival (*P* = 0.000041) (Fig. 7). The patients with the poorest prognosis were observed in the subgroup of 119 of 456 (26.1%) patients characterized by association of high *MMP13* and high *ETV4* mRNA expression levels. These data were also confirmed in the NKI breast cancer cohort (*P* = 0.0013) (Additional file 10: Figure S8a). Multivariate analysis using a Cox proportional hazards model was performed to assess the prognostic value for MFS of the parameters found to be significant in univariate analysis (i.e., SBR histological grade, lymph node status, macroscopic tumor size, PR status [Additional file 2: Table S1] and combined *MMP13* and *ETV4* mRNA levels). The prognostic significance of the lymph node status (*P* = 0.000016), macroscopic tumor size (*P* = 0.0028), and combined *MMP13* and *ETV4* mRNA level was maintained (Additional file 11: Table S2).

We sought links between the three prognostic groups and classical clinicopathological parameters in breast cancer (Table 1). Using HR (ERα and PR) and ERBB2 status, we also subdivided the total population (*n* = 456) into four breast cancer molecular subtypes: HR+/ERBB2+ (*n* = 54), HR+/ERBB2− (*n* = 289), HR−/ERBB2+ (*n* = 45) and HR−/ERBB2− (*n* = 68). High *MMP13* and *ETV4* mRNA expression levels were associated with negative ER status (*P* = 0.00067) and the HR−/ERBB2+ subtype (*P* = 0.0015), two parameters associated with breast cancer aggressiveness (Table 1). We did not observe a correlation between the three prognostic groups and mutations of *PIK3CA*, which is the most frequently mutated oncogene in breast cancer (*P* = 0.96), as well as mRNA level of the *MKI67* gene, which encodes for the proliferation-related Ki-67 antigen (*P* = 0.073).

**Table 1:** Relationship between *MMP13* and *ETV4* transcripts levels and classical clinical biological parameters in 456 breast cancer samples

		Number of patients (%)			
	Total population (%)	Low *ETV4*	High *ETV4*-Low *MMP13*	High *ETV4*-High *MMP13*	*P* value^a^
Total	456 (100.0)	82 (18.0)	255 (55.9)	119 (26.1)	
Age, yr					
≤ 50	98 (21.5)	10 (12.2)	60 (23.5)	28 (23.5)	0.075 (NS)
> 50	358 (78.5)	72 (87.8)	195 (76.5)	91 (76.5)	
SBR histological grade^b, c^					
I	58 (13.0)	10 (12.3)	37 (15.0)	11 (9.2)	0.24 (NS)
II	229 (51.2)	48 (59.3)	122 (49.4)	59 (49.6)	
III	160 (35.8)	23 (28.4)	88 (35.6)	49 (41.2)	
Lymph node status^d^					
0	119 (26.1)	21 (25.9)	67 (26.5)	31 (26.3)	0.085 (NS)
1–3	237 (52.1)	37 (45.7)	144 (56.9)	56 (47.5)	
> 3	96 (21.8)	23 (28.4)	42 (16.6)	31 (26.3)	
Macroscopic tumor size^e^					
≤ 25 mm	223 (49.8)	40 (50.0)	132 (52.4)	51 (44.0)	0.32 (NS)
> 25 mm	225 (50.2)	40 (50.0)	120 (47.6)	65 (56.0)	
ERα status					
Negative	118 (25.9)	9 (11.0)	67 (26.3)	42 (35.3)	**0.00067**
Positive	338 (74.1)	73 (89.0)	188 (73.7)	77 (64.7)	
PR status					
Negative	194 (42.5)	31 (37.8)	103 (40.4)	60 (50.4)	0.12 (NS)
Positive	262 (57.5)	51 (62.2)	152 (59.6)	59 (49.6)	
ERBB2 status					
Negative	357 (78.3)	71 (86.6)	200 (78.4)	86 (72.3)	0.052 (NS)
Positive	99 (21.7)	11 (13.4)	55 (21.6)	33 (27.7)	
Molecular subtypes					
HR−ERBB2−	68 (14.9)	8 (9.8)	41 (16.1)	19 (16.0)	**0.0015**
HR−ERBB2+	45 (9.9)	1 (1.2)	22 (8.6)	22 (18.5)	
HR+ERBB2−	289 (63.4)	63 (76.8)	159 (62.4)	67 (56.3)	
HR+ERBB2+	54 (11.8)	10 (12.2)	33 (12.9)	11 (9.2)	
*PIK3CA* mutation status					
Wild type	307 (67.3)	56 (68.3)	172 (67.5)	79 (66.4)	0.96 (NS)
Mutated	149 (32.7)	26 (31.7)	83 (32.5)	40 (33.6)	
MKI67 mRNA expression					
Median	12.5 (0.80–117)	11.7 (1.74–117)	12.1 (0.80–94.5)	13.6 (2.1–58.5)	0.073 (NS)

*Abbreviations: ERα* Estrogen receptor alpha, *PR* Progesterone receptor, *ERBB2* Human epidermal growth factor receptor 2, *HR* Hormone receptor, *PIK3CA* Phosphatidylinositol-4,5-bisphosphate 3-kinase catalytic subunit alpha, *MKI67* Marker of proliferation Ki-67

The bold values are statistically significant (*P* < 0.05)

Numbers represent the part of the 456 patients in each condition (e.g., age, SBR histological grade) and in regard to the expression level group (Low *ETV4*/High *ETV4*-Low *MMP13*/High *ETV4*-High *MMP13*). For these three groups, percentages in brackets correspond to the proportion of patients in the group (82 for Low *ETV4*; 255 for High *ETV4*-Low *MMP13*; 119 for High *ETV4*-High *MMP13*)

^a^ χ^2^ test

^b^Scarff Bloom Richardson classification

^c^Information available for 447 patients

^d^Information available for 452 patients

^e^Information available for 448 patients

## Discussion

ETV4 is an ETS transcription factor involved in important steps of organ development, such as in mammary gland morphogenesis. ETV4 is also a significant mediator of tumorigenesis through the activation of several downstream pathways that are associated with migration and invasion. ETV4 is overexpressed in breast tumors and is associated with distant metastasis and poor prognosis [1, 5, 39, 40]. However, the cellular and molecular events regulated by this factor remain poorly understood. We previously identified target genes implicated in phenotypic cellular modulation induced in mammary tumorigenesis as Bax or cyclin D2. We have described cyclin D2 to act as a negative regulator of the ETV4-induced responses in mammary cancer cells [8, 12]. We also described that ETV4 overexpression in a mammary epithelial cell line confers tumorigenesis-like properties as well as an increased ability to grow [32] and that ETV4 repression reduces tumorigenesis in mammary cancer cells [16].

In this work, we demonstrate that ETV4 enhances tumorigenic properties of mammary epithelial cancer cells (MMT cells) and that MMP13, as an ETV4 target gene, relays these effects. ETV4 is now well known to be involved in events participating in tumor development and progression. For example, repression of ETV4 in colorectal carcinoma cells significantly impairs their invasive capacity [41], and several EMT markers and MMPs were downregulated in shETV4-expressing cells. In the same way, in gastric adenocarcinoma cell lines, ETV4 increases MMP1 and MMP7 expression and stimulates invasion in vitro [42]. Ectopic overexpression of ETV4 in nonmetastatic human breast cancer cells increases their invasiveness and their metastatic potential in nude mice [43]. Therefore, deregulated metalloprotease expression and/or activity have often been associated with ETV4 tumorigenic properties [1, 44]. However, the precise molecular mechanism by which they act during mammary tumorigenesis is currently unknown.

MMT cells, a mammary tumorigenic cell model, have previously been used to explore the functional involvement of ETV4 in their tumorigenic properties [16]. ETV4 downregulation in MMT cells leads to a decrease in their tumor-forming abilities. Similarly, we show that ETV4 overexpression in MMT cells promotes cell proliferation, migration, invasion, and anchorage-independent growth, demonstrating that ETV4 is an actor of tumorigenic development, as previously described.

Among the well-known MMPs associated with tumorigenic occurrences, MMP13 is a metalloprotease playing an important role in tissue remodeling during fetal and subsequent postnatal bone development [45, 46]. Nevertheless, MMP13 was first identified in a breast tumor library [17], and an increasing amount of data demonstrates its role in tumorigenesis and particularly in breast cancer [22, 24, 27, 47]. In accordance with this, on the basis of transcriptomic analysis, we initially described regulation of MMP13 expression by ETV4 in a mammary epithelial cell line [16].

To shed light on the functional relevance of the ETV4-MMP13 interplay, we explored MMP13 expression in different contexts of murine (TAC, MMT) or human (MCF10A) ETV4-expressing cells and analyzed the regulation of the *MMP13* gene by ETV4. MMP13 expression and activity are positively correlated with ETV4 expression. Moreover, ETV4 is a transactivator of the *MMP13* gene promoter because we identified a 91-bp minimal promoter that contains putative ETS and AP-1 binding sites. We detected the binding of ETV4 to this chromatin region, the cooperation between ETV4 and AP-1 to enhance the transactivation effect of ETV4 and the importance of the proximal EBS, and the requirement of the AP-1 binding motif, a known cofactor of various ETS proteins [43, 48–51]. This synergistic action between AP-1 and ETV4 in MMP13 regulation could emphasize the role of MMP13 in the ETV4-dependent tumorigenic effects and serve as a potential target to treat ETV4-driven diseases.

Given that MMPs are key actors of the tumorigenic and metastatic processes, we evaluated the influence of MMP13 on phenotypic modification of mammary cancer cells and in a context of ETV4 overexpression. On the one hand, MMP13 overexpression is able to slightly increase cell proliferation, migration, and anchorage-independent growth, and on the other hand, MMP13 repression has the reverse effect. In fact, MMP13 has the same behavior as ETV4 in these cancer cells but is less potent. These are relevant findings, considering that MMP13 is overexpressed in a variety of malignant tumors, such as in breast carcinomas [20, 52, 53], and is implicated in bone metastasis in breast cancer [23, 54, 55].

In order to determine if MMP13 is a relay of ETV4 tumorigenic activity, we compared the behavior of MMT cells overexpressing ETV4 and at the same time have a downregulation of MMP13 expression and activity. These ETV4-overexpressing/MMP13-silencing cells show a significant decrease of their proliferation, migration, and anchorage-independent growth rate. Furthermore, we provide evidence that the silencing of MMP13 inhibits ETV4-induced tumor formation in mice, confirming the in vitro data and highlighting the importance of MMP13 activity in ETV4 tumorigenic functions.

Even though numerous studies suggested the importance of MMP13 in tumor progression and metastasis development, by describing its up- or downregulation, very few of them analyzed the impact of these modulations. One of them, by using a similar approach to studying the role of Pit1, a POU class 1 homeobox 1 transcription factor, revealed that it regulates MMP13 expression in human breast cancer cells and that MMP13 knock-down blocks cancer cell invasion into the lungs, suggesting that MMP13 is a mediator of Pit1 induction of breast cancer lung metastasis [56]. These data underline the importance of MMP13 in the mediation of tumorigenesis and invasiveness and corroborate our findings.

The MMT cell model was considered to be a useful model in which to perform the in vitro and in vivo phenotypic assays according to the previously published data and characterization we obtained regarding their ability to form tumors in immunodeficient mice [8, 16]. Indeed, to decipher the relevance of ETV4 and MMP13 association in breast cancer, we assessed *MMP13* and *ETV4* mRNA expression levels in a series of 456 breast cancer samples. Even if high *ETV4* mRNA expression was not shown to be associated with poor MFS, the group with a high *MMP13* mRNA expression level was significantly associated with a bad prognosis (*P* = 0.00016). Nevertheless, by combining *MMP13* and *ETV4* mRNA expression status, we identified three distinct prognostic groups with significantly different MFS curves (*P* = 0.000041). These data revealed that the tumor group overexpressing both *ETV4* and *MMP13* is correlated with the poorest prognosis, much more significant than that of *MMP13* alone. These results were confirmed in the NKI breast cancer cohort (*P* = 0.0013), reinforcing the high prognostic value of *ETV4*- and *MMP13*-associated high expression. Moreover, this correlation was strengthened by the independent prognostic value shown for combined high expression levels of *ETV4* and *MMP13* (*P* = 0.000041). Indeed, our study suggests an important interplay of ETV4 and MMP13 in human breast cancers that could, together, be assessed for their possible signature for guiding diagnosis or therapeutics.

*ETV4* overexpression is associated with increased metastatic risk and poor patient survival in triple-negative breast cancer distant metastasis and poor patient survival [57]. Similarly, high levels of *MMP13* expression are associated with high tumor aggressiveness and poor survival rate [58]. Thus, these data corroborate our findings, and in combination, they underline the importance of these two factors, ETV4 and its relay MMP13, in mammary tumorigenesis. Nevertheless, the real way by which they interplay needs to be deciphered, and further investigations should be done to evaluate their potential as prognostic and diagnostic markers as well as potential therapeutic targets to prevent or treat the disease.

## Conclusions

The ETV4 transcription factor is involved in tumorigenesis and metastatic processes, particularly in breast cancer, a heterogeneous illness with different subtypes. In the present study, we showed that ETV4 promotes proliferation, migration, invasion, and anchorage-independent growth of mammary tumorigenic MMT cells. In parallel, we identified MMP13, an extracellular metalloprotease, as an ETV4 target gene. We showed that, by overexpressing or repressing MMP13 expression, this metalloprotease contributes to ETV4-induced proliferation, migration, and clonogenicity capacity. Thus, MMP13 acts as a relay of ETV4 in its functional role in the mammary epithelial tumorigenic cells in vitro as well as in tumor development in animal models. Finally, we showed that ETV4 and MMP13 co-overexpression is correlated with poor prognosis in breast cancer. Taken together, these data highlight the role of these actors in mammary tumorigenesis and breast cancer progression and underline the potential prognostic value of their combined expression in breast cancer.

## Additional files

Additional file 1: Supplementary methods. (PDF 40 kb)

Additional file 2: **Table S1.** Pathological and clinical characteristics of patients in relation to metastasis-free survival (MFS). (PDF 33 kb)

Additional file 3: **Figure S1.** Validation of the overexpression of ETV4 and MMP13 in MCF10A cells. a and b Relative *ETV4* mRNA (a) and *MMP13* mRNA (b) expression in the MCF10A-Ctrl and MCF10A-ETV4 cells determined by real-time PCR and normalized to cyclophilin A levels. mRNA expression in MCF10A-Ctrl cells was arbitrarily = 1. Error bars indicate SD. *P ≤ 0.1. c and d Western blot analysis of ETV4 protein expression (61 kDa) (c) and MMP13 protein expression (60 kDa) (d) in the MCF10A-Ctrl and MCF10A-ETV4 cells. GAPDH expression served as the loading control. (PDF 100 kb)

Additional file 4: **Figure S2.** ETS and AP-1 binding sites are highly conserved among mouse, human, and rabbit. Nucleotide sequence comparison of mouse, human, and rat proximal *MMP13* promoters. Shaded boxes indicate the conserved ETS and AP-1 binding site sequences. (PDF 46 kb)

Additional file 5: **Figure S3.** ChIP experiment for ETV4 and MMP13 in MMT and TAC cells. PCR detection of the *MMP13* promoter region after ETV4 immunoprecipitation in MMT-ETV4 (left panel) and TAC-ETV4 (right panel). Primers allowing the amplification of the proximal *MMP13* promoter region containing EBS are schematized in the lower panel of Fig. 2. Cyclin D2 was used as a positive control [8]. Immunoprecipitation with a nonrelevant antibody (IgG) was used as negative control. (PDF 60 kb)

Additional file 6: **Figure S4.** Expression of MMP13 in MMT cells overexpressing or repressing MMP13. a and b Relative *MMP13* mRNA expression in the MMT-Ctrl and MMT-MMP13 (a) or MMT-shCtrl and MMT-shMMP13 cells (b) determined by real-time PCR and normalized to cyclophilin A levels. mRNA expression in MMT-Ctrl cells was arbitrarily = 1. Error bars indicate SD. ****P ≤ 0.0001. c Western blot analysis of MMP13 protein expression (60 kDa) in the MMT-Ctrl and MMT-MMP13 cells. GAPDH expression served as the loading control. d Zymographic analysis of MMP13 protein activity (55 kDa) from the supernatant of MMT-Ctrl and MMT-MMP13 cells. (PDF 72 kb)

Additional file 7: **Figure S5.** Expression of ETV4 in MMT-shMMP13-repressing cells. a Relative *ETV4* mRNA expression in the MMT-ETV4 + shCtrl and MMT-ETV4 + shMMP13 cells determined by real-time PCR and normalized to cyclophilin A levels. mRNA expression in MMT-Ctrl + shCtrl cells was arbitrarily = 1. Error bars indicate SD. The results were not statistically significant. b Western blot analysis of ETV4 protein expression (61 kDa) in the MMT-ETV4 + shCtrl and MMT-ETV4 + shMMP13 cells. GAPDH expression served as the loading control. (PDF 71 kb)

Additional file 8: **Figure S6.** The repression of MMP13 reduces the anchorage-independent growth capacity of MMT-ETV4-overexpressing cells. a Relative *MMP13* mRNA expression in the transiently transfected MMT-siCtrl and MMT-siMMP13 cells determined by real-time PCR and normalized to cyclophilin A levels. mRNA expression in MMT-siCtrl cells was arbitrarily = 1. Error bars indicate SD. ****P ≤ 0.0001. b Anchorage-independent growth. MMT-ETV4-siCtrl and MMT-ETV4-siMMP13 cells were cultured for 10 days in soft agar. This histogram represents the number of clones counted for experimental time points. Soft agar assays were conducted three times in triplicate. Magnification × 5. Error bars indicate SD. ****P ≤ 0.0001. (PDF 45 kb)

Additional file 9: **Figure S7.** High *MMP13* mRNA expression level is associated with a poor prognosis in breast cancer. a Metastasis-free survival (MFS) curves for patients with breast tumors according to Low-*MMP13* (*n* = 321) or High-*MMP13* (*n* = 135) mRNA levels. ***P ≤ 0.001. b Metastasis-free survival (MFS) curves for patients with breast tumors according to Low-*ETV4* (*n* = 82) and High-*ETV4* (*n* = 374) mRNA levels. The results were not statistically significant. (PDF 14 kb)

Additional file 10: **Figure S8.** Metastasis-free survival analysis from the publicly available NKI datasets of breast tumors. a Metastasis-free survival (MFS) curves for patients with breast tumors according to Low-*ETV4* (*n* = 12), High-*ETV4* and Low-*MMP13* (*n* = 243), or High-*ETV4* and High-*MMP13* (*n* = 9) mRNA levels. ****P ≤ 0.0001. b Metastasis-free survival (MFS) curves for breast tumor patients according to Low-*MMP13* (*n* = 255) or High-*MMP13* (*n* = 9) mRNA levels. ****P ≤ 0.0001. c Metastasis-free survival (MFS) curves for patients with breast tumors according to Low-*ETV4* (*n* = 13) and High-*ETV4* (*n* = 251) mRNA levels. ****P ≤ 0.0001. (PDF 19 kb)

Additional file 11: **Table S2.** Multivariate Cox proportional hazards analysis of MFS for *MMP13* and *ETV4* expression levels in the series of 456 breast tumors. (PDF 42 kb)

## Abbreviations

AP-1: Activator protein 1
bp: base pair
BSA: Bovine serum albumin
cDNA: Complementary DNA
ChIP: Chromatin immunoprecipitation
EBS: ETS binding site
EMT: Epithelial-mesenchymal transition
ERBB2: human epidermal growth factor receptor 2
ERα: Estrogen receptor alpha
ETV-4: ETS translocation variant 4
GAPDH: Glyceraldehyde 3-phosphate dehydrogenase
HEK-293: Human embryonic kidney 293 cells
HR: Hormone receptor
HRP: Horseradish peroxidase
IgG: Immunoglobulin G
MFS: Metastasis-free survival
MMP: Matrix metalloproteinase
MMT: Mouse mammary tumor
mRNA: Messenger RNA
PET: Polyethylene terephthalate
PIK3CA: Phosphatidylinositol 3-kinase catalytic
PR: Progesterone receptor
SBR: Scarff-Bloom-Richardson histoprognostic grading system
SCID: Severe combined immunodeficiency
shRNA: Short hairpin RNA
siRNA: Small interfering RNA
TNBC: Triple-negative breast cancer
