# Supplementary material for: ETV4 transcription factor and MMP13 metalloprotease are interplaying actors of breast tumorigenesis
Source: Breast Cancer Res. 2018 Jul 11;20:73. doi: 10.1186/s13058-018-0992-0 (PMC6042225; doi:10.1186/s13058-018-0992-0)
Supplement: Supplementary file 1 — Supplementary methods. (PDF 40 kb) [file 13058_2018_992_MOESM1_ESM.pdf]

Supplementary Methods

Chromatin Immuno-Precipitation (ChIP) detailed protocol

MMT or TAC cells were fixed for 10 minutes with a 1.1% formaldehyde solution at room temperature. Fixation was quenched by the addition of 2.5 M glycin to a 150 mM final concentration. Cells were then washed with cold PBS and lysed in 450 µl of Lysis buffer (20 mM Tris-HCl pH8.1, 10 mM EDTA pH8.0, 1% SDS). Chromatin was sheared using a Bioruptor Sonicator set to high power with cycles of 30 sec on 30 sec off for a total time of 10 minutes (Diagenode). The cell lysate was then diluted ten folds in IP buffer (20 mM Tris-HCl pH8.1, 2 mM EDTA pH8.0, 150 mM NaCl, 1% Triton X-100) and pre- cleared during 3 hours by the addition of 400 µl of a 50% protein G agarose beads slurry. The protein complexes were then immuno-precipitated by the addition of 4 µg of an anti-ETV4 IgG (sc-113X, Santa Cruz) or a non-relevant antibody (anti-Bax sc-7480) to the pre- cleared lysate overnight at 4°C and addition of 180 µl of a 50% protein G agarose beads slurry for 4 hours at 4°C. The beads were then washed with buffers TSEI (20 mM Tris-HCl pH8.1, 2 mM EDTA pH8.0, 150 mM NaCl, 1% Triton X-100, 0.1% SDS), TSEII (20 mM Tris- HCl pH8.1, 2 mM EDTA pH8.0, 500 mM NaCl, 0.1% Triton X-100, 0.1% SDS), Buffer 3 (10m M Tris-HCl pH8.1, 1 mM EDTA pH8.0, 1% NP-40, 250 mM LiCl, 1% deoxycholate) and twice with TE (10 mM Tris-HCl pH8.1, 1 mM EDTA pH8.0). DNA-Protein complexes were then eluted by incubation of the beads in elution buffer (1% SDS, 100 for 15 minutes. Cross-linking was reversed by the addition of 5 M Sodium Chloride to a 200 mM final concentration and incubation at 65°C overnight. Finally, the DNA fragments were purified using the Wizard® Genomic DNA Purification Kit (Promega).

Real-time qRT-PCR for MMP13 and ETV4 expressions

Quantitative values were obtained from the cycle number (Ct value) at which the increase in the fluorescence signal associated with exponential growth of PCR products started to be detected by the laser detector of the ABI Prism 7900 sequence detection system (Perkin–

Elmer Applied Biosystems, Foster City, CA), using PE biosystems analysis software according to the manufacturer's manuals.

The precise amount of total mRNA added to each reaction mix (based on optical density) and its quality (i.e., lack of extensive degradation) are both difficult to assess. Therefore, we also quantified transcripts of the TBP gene (Genbank accession NM_003194) encoding the TATA box-binding protein (a component of the DNA-binding protein complex TFIID) as an endogenous RNA control, and normalized each sample on the basis of its TBP content. We selected TBP as an endogenous control because the prevalence of its transcripts is moderate, and because there are no known TBP retropseudogenes (retropseudogenes lead to co-amplification of contaminating genomic DNA and thus interfere with RT-PCR, despite the use of primers in separate exons) [1, 2].

Results, expressed as N-fold differences in target (MMP13 or ETV4) genes expressions relative to the TBP gene and termed ''NTARGET'' were determined as NTARGET = 2ΔCtsample, where the ΔCt value of the sample was determined by subtracting the Ct value of the target gene from the Ct value of the TBP gene.

The NTARGET values of the samples were subsequently normalized such that the median of the NTARGET values for the 10 normal breast tissues was 1.

The primers for MMP13, ETV4 and TBP were chosen with the assistance of the Oligo 6.0 program (National Biosciences, Plymouth, MN). We scanned the dbEST and nr databases to confirm the total gene specificity of the nucleotide sequences chosen for the primers and the absence of single nucleotide polymorphisms. The nucleotide sequences of the primers used were as follows: MMP13-U (5'-AGGAAACCAGGTCTGGAGATATGAT-3') and MMP13-L (5'-CCAATTCCTGGGAAGTCTTCTTCT-3') for MMP13 gene (PCR product of 90 bp), ETV4-U (5'-AGGAGACGTGGCTCGCTG-3') and ETV4-L (5'-GGGGCTGTGGAAAGCTAGGTT-3') for ETV4 gene (PCR product of 92 bp), and TBP-U (5'-TGCACAGGAGCCAAGAGTGAA-3') and TBP-L (5'-CACATCACAGCTCCCCACCA-3') for TBP gene (PCR product of 132 bp). To avoid amplification of contaminating genomic DNA, one of the two primers was placed at the

junction between two exons. Agarose gel electrophoresis was used to verify the specificity of PCR amplicons.

1. Bieche I, Onody P, Laurendeau I, Olivi M, Vidaud D, Lidereau R, Vidaud M (1999) Real-time reverse transcription-PCR assay for future management of ERBB2-based clinical applications. Clin Chem. 1999 Aug;45(8 Pt 1):1148-56.
2. Bieche I, Parfait B, Laurendeau I, Girault I, Vidaud M, Lidereau R (2001) Quantification of estrogen receptor alpha and beta expression in sporadic breast cancer. Oncogene. 2001 Dec 6;20(56):8109-15.
