# Supplementary material for: ETV4 transcription factor and MMP13 metalloprotease are interplaying actors of breast tumorigenesis
Source: Breast Cancer Res. 2018 Jul 11;20:73. doi: 10.1186/s13058-018-0992-0 (PMC6042225; doi:10.1186/s13058-018-0992-0)
Supplement: Supplementary file 2 — Table S1. Pathological and clinical characteristics of patients in relation to metastasis-free survival (MFS). (PDF 33 kb) [file 13058_2018_992_MOESM2_ESM.pdf]

**Table S1: Pathological and clinical characteristics of patients in relation to metastasis free survival (MFS)**

| | Number of patients (%) | Number of metastasis (%) | MFS *p*-value [a] |
|---|---|---|---|
| *Total* | 456 (100.0) | 181 (39.7) | |
| *Age* | | | |
| ≤50 | 98 (21.5) | 39 (39.8) | 0.88 (NS) |
| >50 | 358 (78.5) | 142 (39.7) | |
| *SBR histological grade* [b, c] | | | |
| I | 58 (13) | 11 (19.0) | **0.0011** |
| II | 229 (51.2) | 93 (40.6) | |
| III | 160 (35.8) | 73 (45.6) | |
| *Lymph node status* [d] | | | |
| 0 | 119 (26.1) | 36 (30.3) | **0.00000012** |
| 1-3 | 237 (52.1) | 83 (35.0) | |
| >3 | 96 (21.8) | 60 (62.5) | |
| *Macroscopic tumor size* [e] | | | |
| ≤25mm | 223 (49.8) | 69 (30.9) | **0.000014** |
| >25mm | 225 (50.2) | 111 (49.3) | |
| *ER$\alpha$ status* | | | |
| Negative | 118 (25.9) | 50 (42.4) | 0.077 (NS) |
| Positive | 338 (74.1) | 131 (38.8) | |
| *PR status* | | | |
| Negative | 194 (42.5) | 86 (44.3) | **0.013** |
| Positive | 262 (57.5) | 95 (36.3) | |
| *ERBB2 status* | | | |
| Negative | 357 (78.3) | 138 (38.7) | 0.27 (NS) |
| Positive | 99 (21.7) | 43 (43.4) | |
| *Molecular subtypes* | | | |
| HR- ERBB2- | 68 (14.9) | 26 (38.2) | 0.055 (NS) |
| HR- ERBB2+ | 45 (9.9) | 23 (51.1) | |
| HR+ ERBB2- | 289 (63.4) | 112 (38.8) | |
| HR+ ERBB2+ | 54 (11.8) | 20 (37.0) | |
| *PIK3CA mutation status* | | | |
| wild type | 307 (67.3) | 129 (42.0) | 0.071 (NS) |
| mutated | 149 (32.7) | 52 (34.9) | |
| *MMP13* and *ETV4* mRNA expression | | | |
| Low *ETV4* | 82 (18.0) | 30 (36.6) | |
| Low *MMP13* and high *ETV4* | 255 (55.9) | 86 (33.7) | **0.000041** |
| High *MMP13* and high *ETV4* | 119 (26.1) | 65 (54.6) | |

Abbreviations: ER$\alpha$: oestrogen receptor alpha; PR: progesterone receptor; ERBB2: human epidermal growth factor receptor 2. The bold values are statistically significant (*p*-value<0.05).

[a] Log-rank test.

[b] Scarff Bloom Richardson classification.

[c] Information available for 447 patients.

[d] Information available for 452 patients.

e Information available for 448 patients.
