# Supplementary figures and images for: ETV4 transcription factor and MMP13 metalloprotease are interplaying actors of breast tumorigenesis
Source: Breast Cancer Res. 2018 Jul 11;20:73. doi: 10.1186/s13058-018-0992-0 (PMC6042225; doi:10.1186/s13058-018-0992-0)

a.

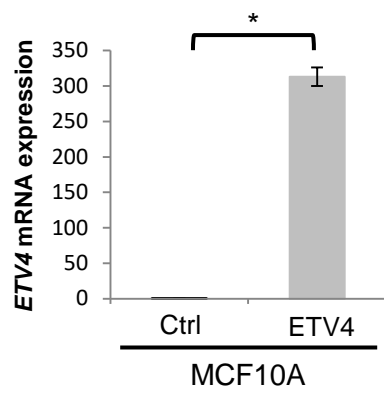

b.

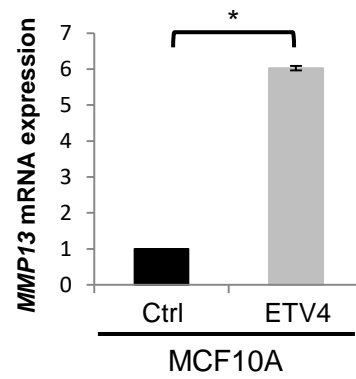

c.

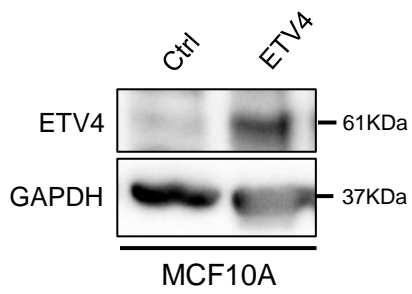

d.

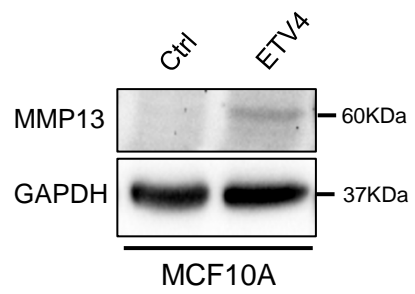

Figure S1

Supplement: Supplementary file 3 — Figure S1. Validation of the overexpression of ETV4 and MMP13 in MCF10A cells. a and b Relative ETV4 mRNA (a) and MMP13 mRNA (b) expression in the MCF10A-Ctrl and MCF10A-ETV4 cells determined by real-time PCR and normalized to cyclophilin A levels. mRNA expression in MCF10A-Ctrl cells was arbitrarily = 1. Error bars indicate SD. *P ≤ 0.1. c and d Western blot analysis of ETV4 protein expression (61 kDa) (c) and MMP13 protein expression (60 kDa) (d) in the MCF10A-Ctrl and MCF10A-ETV4 cells. GAPDH expression served as the loading control. (PDF 100 kb) [file 13058_2018_992_MOESM3_ESM.pdf]

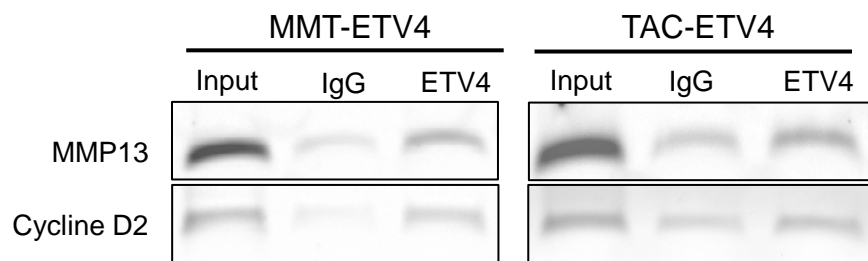

**Figure S3**

Supplement: Supplementary file 5 — Figure S3. ChIP experiment for ETV4 and MMP13 in MMT and TAC cells. PCR detection of the MMP13 promoter region after ETV4 immunoprecipitation in MMT-ETV4 (left panel) and TAC-ETV4 (right panel). Primers allowing the amplification of the proximal MMP13 promoter region containing EBS are schematized in the lower panel of Fig. 2. Cyclin D2 was used as a positive control [8]. Immunoprecipitation with a nonrelevant antibody (IgG) was used as negative control. (PDF 60 kb) [file 13058_2018_992_MOESM5_ESM.pdf]

a.

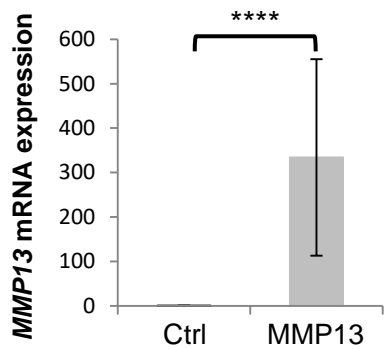

b.

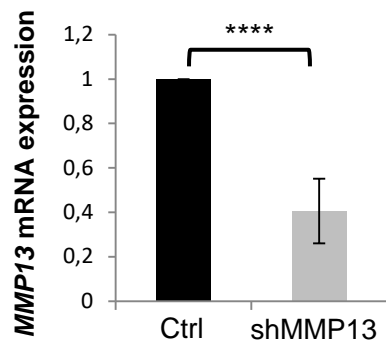

c.

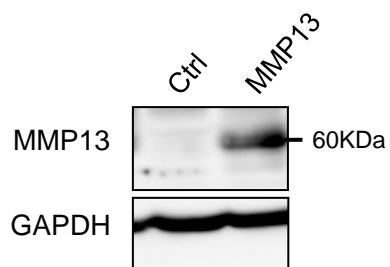

d.

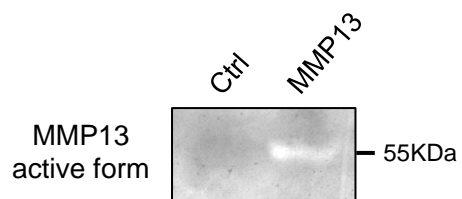

Figure S4

Supplement: Supplementary file 6 — Figure S4. Expression of MMP13 in MMT cells overexpressing or repressing MMP13. a and b Relative MMP13 mRNA expression in the MMT-Ctrl and MMT-MMP13 (a) or MMT-shCtrl and MMT-shMMP13 cells (b) determined by real-time PCR and normalized to cyclophilin A levels. mRNA expression in MMT-Ctrl cells was arbitrarily = 1. Error bars indicate SD. ****P ≤ 0.0001. c Western blot analysis of MMP13 protein expression (60 kDa) in the MMT-Ctrl and MMT-MMP13 cells. GAPDH expression served as the loading control. d Zymographic analysis of MMP13 protein activity (55 kDa) from the supernatant of MMT-Ctrl and MMT-MMP13 cells. (PDF 72 kb) [file 13058_2018_992_MOESM6_ESM.pdf]

a.

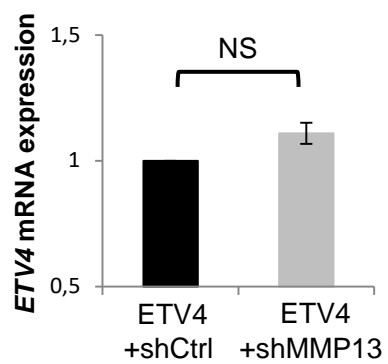

b.

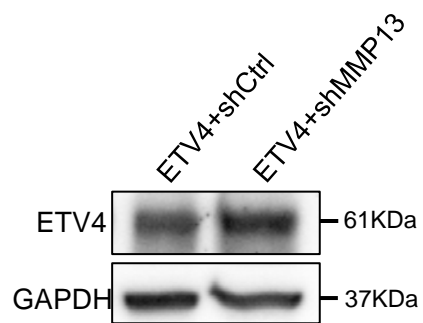

Figure S5

Supplement: Supplementary file 7 — Figure S5. Expression of ETV4 in MMT-shMMP13-repressing cells. a Relative ETV4 mRNA expression in the MMT-ETV4 + shCtrl and MMT-ETV4 + shMMP13 cells determined by real-time PCR and normalized to cyclophilin A levels. mRNA expression in MMT-Ctrl + shCtrl cells was arbitrarily = 1. Error bars indicate SD. The results were not statistically significant. b Western blot analysis of ETV4 protein expression (61 kDa) in the MMT-ETV4 + shCtrl and MMT-ETV4 + shMMP13 cells. GAPDH expression served as the loading control. (PDF 71 kb) [file 13058_2018_992_MOESM7_ESM.pdf]

a.

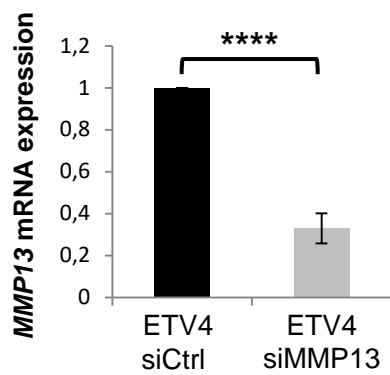

b.

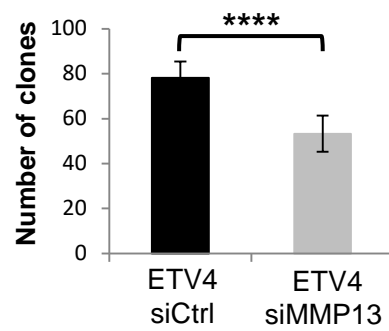

Figure S6

Supplement: Supplementary file 8 — Figure S6. The repression of MMP13 reduces the anchorage-independent growth capacity of MMT-ETV4-overexpressing cells. a Relative MMP13 mRNA expression in the transiently transfected MMT-siCtrl and MMT-siMMP13 cells determined by real-time PCR and normalized to cyclophilin A levels. mRNA expression in MMT-siCtrl cells was arbitrarily = 1. Error bars indicate SD. ****P ≤ 0.0001. b Anchorage-independent growth. MMT-ETV4-siCtrl and MMT-ETV4-siMMP13 cells were cultured for 10 days in soft agar. This histogram represents the number of clones counted for experimental time points. Soft agar assays were conducted three times in triplicate. Magnification × 5. Error bars indicate SD. ****P ≤ 0.0001. (PDF 45 kb) [file 13058_2018_992_MOESM8_ESM.pdf]

a.

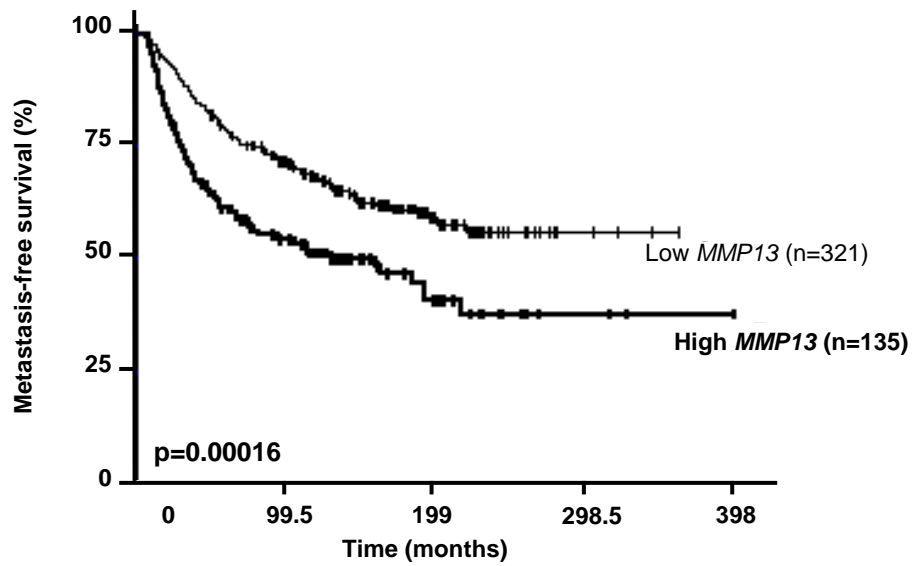

b.

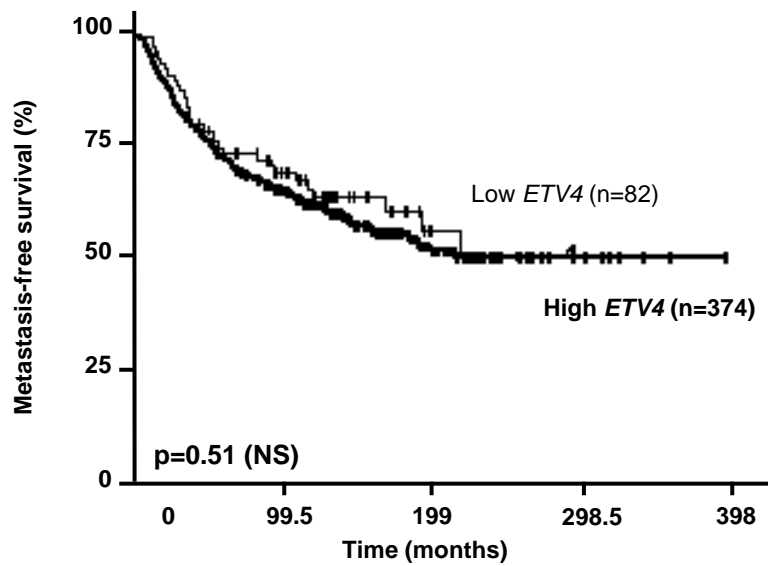

Figure S7

Supplement: Supplementary file 9 — Figure S7. High MMP13 mRNA expression level is associated with a poor prognosis in breast cancer. a Metastasis-free survival (MFS) curves for patients with breast tumors according to Low-MMP13 (n = 321) or High-MMP13 (n = 135) mRNA levels. ***P ≤ 0.001. b Metastasis-free survival (MFS) curves for patients with breast tumors according to Low-ETV4 (n = 82) and High-ETV4 (n = 374) mRNA levels. The results were not statistically significant. (PDF 14 kb) [file 13058_2018_992_MOESM9_ESM.pdf]

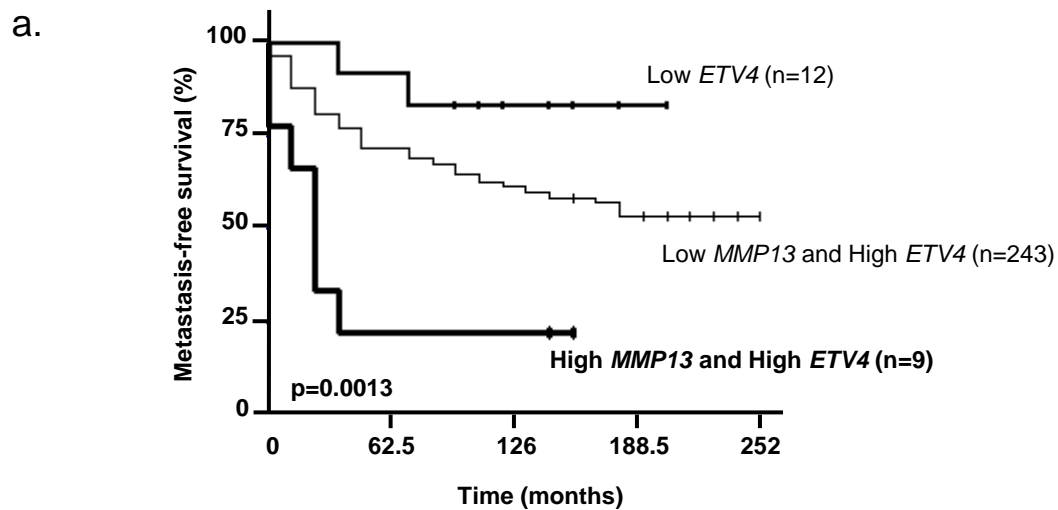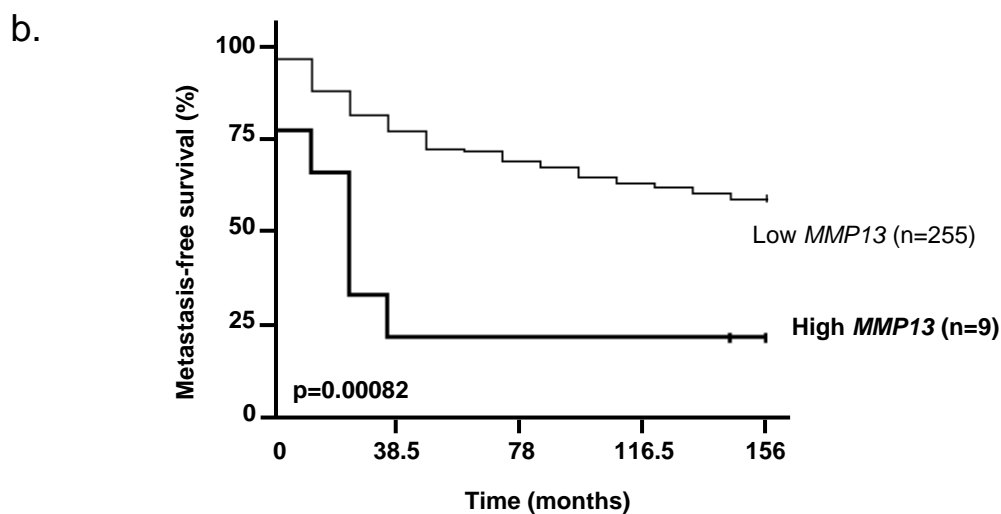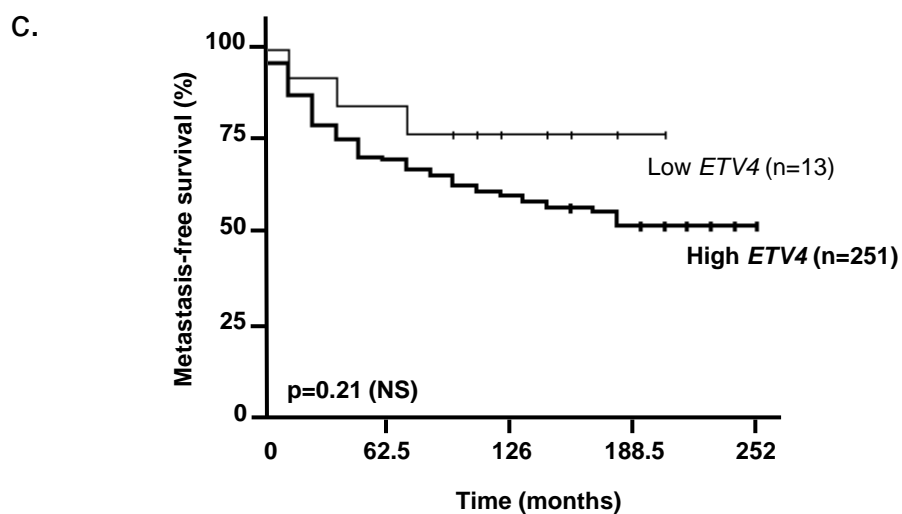

Figure S8

Supplement: Supplementary file 10 — Figure S8. Metastasis-free survival analysis from the publicly available NKI datasets of breast tumors. a Metastasis-free survival (MFS) curves for patients with breast tumors according to Low-ETV4 (n = 12), High-ETV4 and Low-MMP13 (n = 243), or High-ETV4 and High-MMP13 (n = 9) mRNA levels. ****P ≤ 0.0001. b Metastasis-free survival (MFS) curves for breast tumor patients according to Low-MMP13 (n = 255) or High-MMP13 (n = 9) mRNA levels. ****P ≤ 0.0001. c Metastasis-free survival (MFS) curves for patients with breast tumors according to Low-ETV4 (n = 13) and High-ETV4 (n = 251) mRNA levels. ****P ≤ 0.0001. (PDF 19 kb) [file 13058_2018_992_MOESM10_ESM.pdf]
