# Supplementary material for: ETV4 transcription factor and MMP13 metalloprotease are interplaying actors of breast tumorigenesis
Source: Breast Cancer Res. 2018 Jul 11;20:73. doi: 10.1186/s13058-018-0992-0 (PMC6042225; doi:10.1186/s13058-018-0992-0)
Supplement: Supplementary file 4 — Figure S2. ETS and AP-1 binding sites are highly conserved among mouse, human, and rabbit. Nucleotide sequence comparison of mouse, human, and rat proximal MMP13 promoters. Shaded boxes indicate the conserved ETS and AP-1 binding site sequences. (PDF 46 kb) [file 13058_2018_992_MOESM4_ESM.pdf]

```
mus    AAGTAGAGATGCCTTCATTTTCCATTTCCCTCAGATTCTGCCACAAACCACACTTAGGAA
homo   AACAAGAGATGCTCTCATTTAT-ATTTCCCTCAAATTCTACCACAAACCACACTCGGGAG
rattus AACTAGAGATGCCCTAATTTTCCATTTCCCTCAGGTTCTGCCACAAACCACACGTACGAA
       **  ******** * ****   **********  **** *************    **

mus    GAAAAAAAAATA----CCATGTAAGCATGTTTACCTTCGCCTCACTAGGAAGTTAACACA
homo   GGAAAAGAAAAAGTCGCCACGTAAGCATGTTTACCTTCAAGTGACTGGGAAGTGGAAACC
rattus --AAAAAAAATA----CCACGTAAGCATGTTTACCTTCGACTCACTAGGAAGTGAACACC
         **** *** *    *** ******************   * *** ******  * **

mus    CACC--CCAAAGTGGTGACTCATCACTATCATGCTATAAAATAGAAGATG----------
homo   TATC--CATAAGTGATGACTCACCATTGCAGGCCTATAAAAGTAAAGGTAATCTCTGCGG
rattus CCCCACCCCAAGTGGTGACTCATCACTATTGCTCTATAAAATAGAGAATGCTTGCCCTGG
          *  *  ***** ******* ** *      ********   *   *

mus    ---------------------
homo   AAAG-----------------
rattus GAAGGAGAGACTCCAGGCACT
```

ETS Binding Site

AP1 Binding Site

Figure S2
