# Supplementary material for: ETV4 transcription factor and MMP13 metalloprotease are interplaying actors of breast tumorigenesis
Source: Breast Cancer Res. 2018 Jul 11;20:73. doi: 10.1186/s13058-018-0992-0 (PMC6042225; doi:10.1186/s13058-018-0992-0)
Supplement: Supplementary file 11 — Table S2. Multivariate Cox proportional hazards analysis of MFS for MMP13 and ETV4 expression levels in the series of 456 breast tumors. (PDF 42 kb) [file 13058_2018_992_MOESM11_ESM.pdf]

Table S2: Multivariate COX analysis of MFS for *MMP13* and *ETV4* expression level in the series of 456 breast tumors.

**Characteristics**		**HR** [a]	**95% CI** [b]	***p*-value** [c]
Lymph node status	0	1		**0.000016**
	1-3	1.63	1.30-2.03	
	>3	2.64	1.70-4.11	
MMP13 and ETV4 mRNA expression	Low *MMP13* and high *ETV4*	1		**0.00004**
	Low *ETV4*	1.42	1.20-1.67	
	High *MMP13* and high *ETV4*	2.01	1.44-2.80	
Macroscopic tumor size	≤25mm	1		**0.0028**
	>25mm	1.61	1.18-2.21	
SBR histological grade	I	1		
	II	1.23	0.96-1.57	0.11 (NS)
	III	1.5	0.92-2.46	
PR status	Positive	1		
	Negative	1.25	0.91-1.72	0.17 (NS)

[a] Hazard ratio.

[b] 95% Confidential Interval.

[c] Multivariate COX analysis.
